# Endothelial tip cells *in vitro* are less glycolytic and have a more flexible response to metabolic stress than non-tip cells

**DOI:** 10.1038/s41598-019-46503-2

**Published:** 2019-07-18

**Authors:** B. Yetkin-Arik, I. M. C. Vogels, N. Neyazi, V. van Duinen, R. H. Houtkooper, C. J. F. van Noorden, I. Klaassen, R. O. Schlingemann

**Affiliations:** 1 Ocular Angiogenesis Group, Department of Ophthalmology, Amsterdam Cardiovascular Sciences and Cancer Center Amsterdam, Amsterdam UMC, University of Amsterdam, Meibergdreef 9, Amsterdam, The Netherlands; 2 Department of Medical Biology, Amsterdam Cardiovascular Sciences and Cancer Center Amsterdam, Amsterdam UMC, University of Amsterdam, Meibergdreef 9, Amsterdam, The Netherlands; 3 Department of Systems Biomedicine and Pharmacology, Leiden University, Leiden, The Netherlands; 4 Department of Internal Medicine, Division of Nephrology and the Einthoven Laboratory for Vascular and Regenerative Medicine, Leiden University Medical Centre, Leiden, The Netherlands; 5 Laboratory Genetic Metabolic Diseases, Amsterdam Gastroenterology and Metabolism, Amsterdam Cardiovascular Sciences, Amsterdam UMC, University of Amsterdam, Meibergdreef 9, Amsterdam, The Netherlands; 6 Department of Genetic Toxicology and Cancer Biology, National Institute of Biology, Ljubljana, Slovenia; 7 Department of Ophthalmology, University of Lausanne, Jules-Gonin Eye Hospital, Fondation Asile des Aveugles, Lausanne, Switzerland

**Keywords:** Cell growth, Mechanisms of disease, Angiogenesis

## Abstract

Formation of new blood vessels by differentiated endothelial tip cells, stalk cells, and phalanx cells during angiogenesis is an energy-demanding process. How these specialized endothelial cell phenotypes generate their energy, and whether there are differences between these phenotypes, is unknown. This may be key to understand their functions, as (1) metabolic pathways are essentially involved in the regulation of angiogenesis, and (2) a metabolic switch has been associated with angiogenic endothelial cell differentiation. With the use of Seahorse flux analyses, we studied metabolic pathways in tip cell and non-tip cell human umbilical vein endothelial cell populations. Our study shows that both tip cells and non-tip cells use glycolysis as well as mitochondrial respiration for energy production. However, glycolysis is significantly lower in tip cells than in non-tip cells. Additionally, tip cells have a higher capacity to respond to metabolic stress. Finally, in non-tip cells, blocking of mitochondrial respiration inhibits endothelial cell proliferation. In conclusion, our data demonstrate that tip cells are less glycolytic than non-tip cells and that both endothelial cell phenotypes can adapt their metabolism depending on microenvironmental circumstances. Our results suggest that a balanced involvement of metabolic pathways is necessary for both endothelial cell phenotypes for proper functioning during angiogenesis.

## Introduction

Recently, differential activation of metabolic pathways in endothelial cells (ECs) has been identified as a regulatory mechanism in angiogenesis. Angiogenesis occurs in many pathological conditions, in both neovascular eye diseases such as retinopathy of prematurity, age-related macular degeneration, and diabetic retinopathy^1–3^, as in neovascularization of tumors^4–6^. During angiogenesis, ECs shift from a quiescent population to a population of heterogeneous activated phenotypes, each with a distinct cellular differentiation pattern. Firstly, a single tip cell develops, which is the leading cell of a blood vessel sprout that grows out from a pre-existing vascular network into the extracellular matrix, attracted by microenvironmental signals for migration. Secondly, stalk cells follow the tip cell, and these are highly proliferative, produce extracellular matrix, and form a vessel lumen. Thirdly, the phalanx cell is the quiescent EC phenotype that is eventually acquired by differentiated ECs once the new vessel is formed. Many cytokines, growth factors, and other molecules have been identified that regulate tip and stalk cell functions and angiogenesis^7,8^.

In recent publications, specific metabolic pathways have been identified as major regulators of angiogenesis. Metabolism is necessary to fuel vascular expansion and to control the formation of new blood vessels^9^. In animal and *in vitro* models, it was demonstrated that resting ECs in general have a glycolytic phenotype and that ECs as an overall population increase their glycolytic flux in response to angiogenic activation^10,11^. However, the relative contribution and regulatory functions of different metabolic pathways in tip cells and the other angiogenic phenotypes, respectively, could not be determined in these studies, as the models employed do not allow for appropriate discrimination between tip cells and other angiogenic phenotypes.

**Figure 1:**
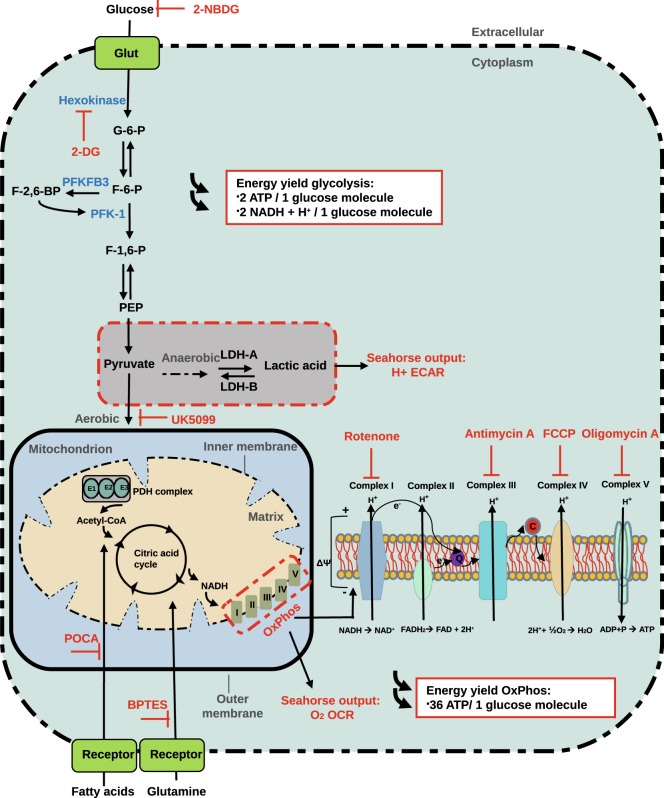
Cellular glycolysis and oxidative phosphorylation. Glycolysis and oxidative phosphorylation are two major energy-yielding pathways. Glucose is converted into pyruvate in the glycolytic pathway. The fate of pyruvate is dependent on many factors, of which oxygen availability is important. In anaerobic conditions, pyruvate is converted into lactate by LDH-A in the cytoplasm. LDH-B converts lactate into pyruvate. ECAR is a measure of lactic acid levels, generated by anaerobic glycolysis. In aerobic conditions, pyruvate enters the citric acid cycle via the PDH complex, and is catabolized by oxidative phosphorylation, producing electrons that pass through the electron transport chain to pump protons across the inner mitochondrial membrane. Protons are accumulated in the mitochondrial intermembrane space, and generates an electrochemical gradient across the inner mitochondrial membrane that is used for ATP production by ATP synthase (complex Ѵ). OCR is a measure of oxygen utilization in cells and is an indicator of mitochondrial function. The conversion of glucose into lactate generates 2 ATP per glucose molecule as compared to 36 ATP per glucose molecule when the oxidative phosphorylation is used. 2-NBDG; 2-[N-(7-nitobenz-2-oxa-1,3-diazol-4-yl)-amino]-2-deoxy-D glucose. 2-DG; 2-deoxyglucose. Glut; glucose transporters. G-6-P; glucose-6-phosphate. F-6-P; fructose-6-phosphate. PFKFB3; 6-phosphofructo-2-kinase/fructose-2,6-biphosphatase 3. F-2,6-BP; fructose-2,6-biphosphate. F-1,6-P; fructose-1,6-phosphate. PEP; phosphoenolpyruvate. LDH-A; lactate dehydrogenase A. LDH-B; lactate dehydrogenase B. PDH; pyruvate dehydrogenase. NADH; nicotinamide adenine dinucleotide. FADH_2_; Flavin adenine dinucleotide. H^+^; proton. OxPhos; oxidative phosphorylation. BPTES; bis-2-2(5-phenylacetamido-1,3,4-thiadiazol-2-yl) ethyl sulfide. POCA; sodium 2-[5-(4-chlorophenyl)pentyl]-oxirane-2-carboxylate. ECAR; extracellular acidification rates. OCR; oxygen consumption rates. ADP; adenosine 5’-diphosphate. ATP; adenosine 5’-triphosphate. FCCP; carbonyl cyanide 4-(trifluoromethoxy) phenylhydrazone.

We have developed an *in vitro* technique to identify and separate CD34^+^ tip cells from CD34^−^ non-tip cells in EC cultures^12^. We have verified the tip cell specificity of these CD34^+^ tip cells by several molecular and cell biological methods, after isolation by fluorescence-assisted cell sorting (FACS). We showed that these cells have an increased abundance of the mRNAs of all known tip cell-specific genes. Genome-wide mRNA profiling analysis of CD34^+^ ECs demonstrated enrichment for biological functions related to angiogenesis and migration, whereas CD34^−^ ECs were enriched for functions related to cell proliferation^12^. Gene set enrichment analysis (GSEA) showed that our tip cell gene set correlated positively with tip cell gene sets from other studies^13–15^. Furthermore, tip cells showed a much lower proliferation rate. Finally, the CD34^+^ phenotype was upregulated by vascular endothelial growth factor A (VEGF-A) and downregulated by tumor necrosis factor-alpha (TNF-α) and delta-like-4 (DLL4), three mechanisms known to regulate the tip cell phenotype *in vivo*^12^. Recently, we reproduced these findings in human microvascular endothelial cells (hMVECs), a more relevant endothelial cell type for angiogenesis^16^. The purpose of the present study was to establish the specific energy metabolism of tip cells as compared to non-tip cells, and to shed light on the potential regulatory roles of metabolic pathways, as shown in Fig. 1, during angiogenesis.

## Results

### Glucose is necessary for energy production and survival of huvecs

Cellular processes during angiogenesis, such as migration, proliferation, and capillary organization, require energy-rich adenosine 5′-triphosphate (ATP). Glucose uptake and metabolism are increased during angiogenesis to meet this energy demand^17^. We tested whether glucose uptake is necessary for energy production and survival of human umbilical vein endothelial cells (HUVECs). The non-metabolizable 2-DG, a glucose analog that inhibits the initial step of glycolysis by its interaction with hexokinase, was used to determine the glucose dependency of HUVECs (Fig. 1). HUVECs were treated with 2-DG in medium containing energy substrates (such as glucose, glutamine, lactate, and fatty acids)^18^, and total ATP levels and cell viability were assessed after 0.5, 1, 4, and 24 h of treatment. Total ATP levels were rapidly reduced after 2-DG treatment and reached a minimum of 25% of baseline levels after 1 h that lasted up to 24 h (Supplementary Fig. S1A). Cell viability was not affected during the first 4 h after treatment, but was reduced by 2.2-fold at 24 h after treatment (Supplementary Fig. S1B).

Overall, these data confirm that glucose is necessary for ATP production and for survival of HUVECs. Whether ATP produced via either glycolysis or mitochondrial respiration is investigated next.

### CD34^+^ cells are mainly present on the tips of sprouts

**Figure 2:**
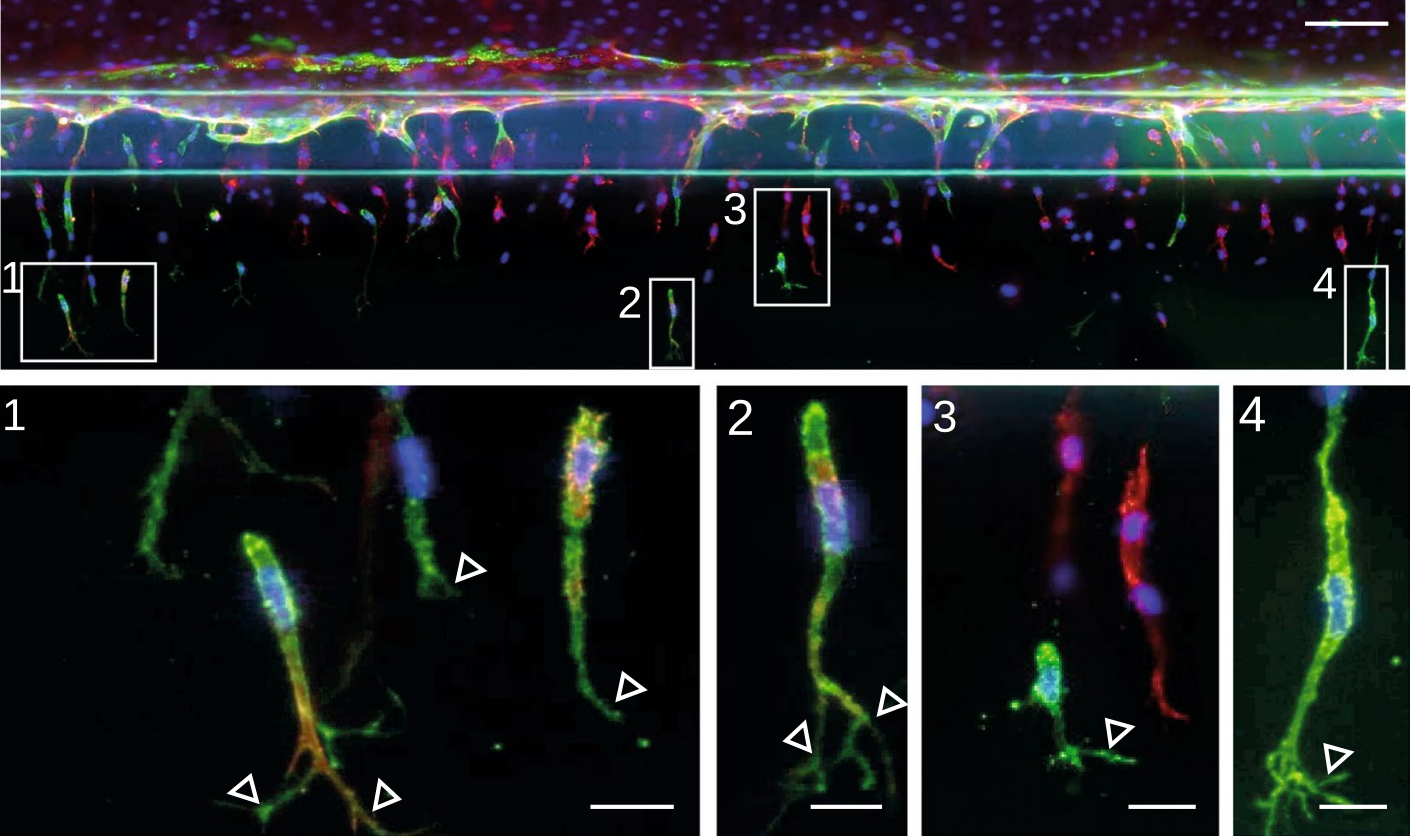
CD34^+^ tip cells in a 3D angiogenic sprouting model. (**a**) Angiogenic sprouts are stained for CD34 (green), F-actin (red) and nuclei (blue). Magnifications are shown and indicated in the overview image. Open triangles point at filopodial extensions. Scale bars, 100 µm (overview) 1-4: 20 µm (magnifications).

Endothelial cells in a monolayer that grow semi-confluent and that are supplemented with serum-containing medium, which is rich in pro-angiogenic growth factors^19^, contain a subset of cells that display a tip cell phenotype and can be identified with anti-CD34 antibody^12,16^. In our previous studies, we provided evidence that CD34^+^ cells are genotypically and phenotypically similar to tip cells *in vitro*, but we did not yet show the existence of these CD34^+^ cells in a sprouting model. For this purpose, we used a 3D angiogenesis model^20^. After the formation of a confluent microvessel, sprouting was induced by the addition of VEGF, spingosine-1-phosphate (S1P) and phorbol 12-myristate 13-acetate (PMA). Sprouts were stained for CD34 and F-actin, and we observed that CD34 staining was mainly present in cells on the tips of sprouts that have a tip cell morphology including filopodial extensions (Fig. 2). This finding confirms that CD34 is a marker for tip cells *in vitro*.

### VEGF is necessary for directional movement of CD34^+^ tip cells

**Figure 3:**
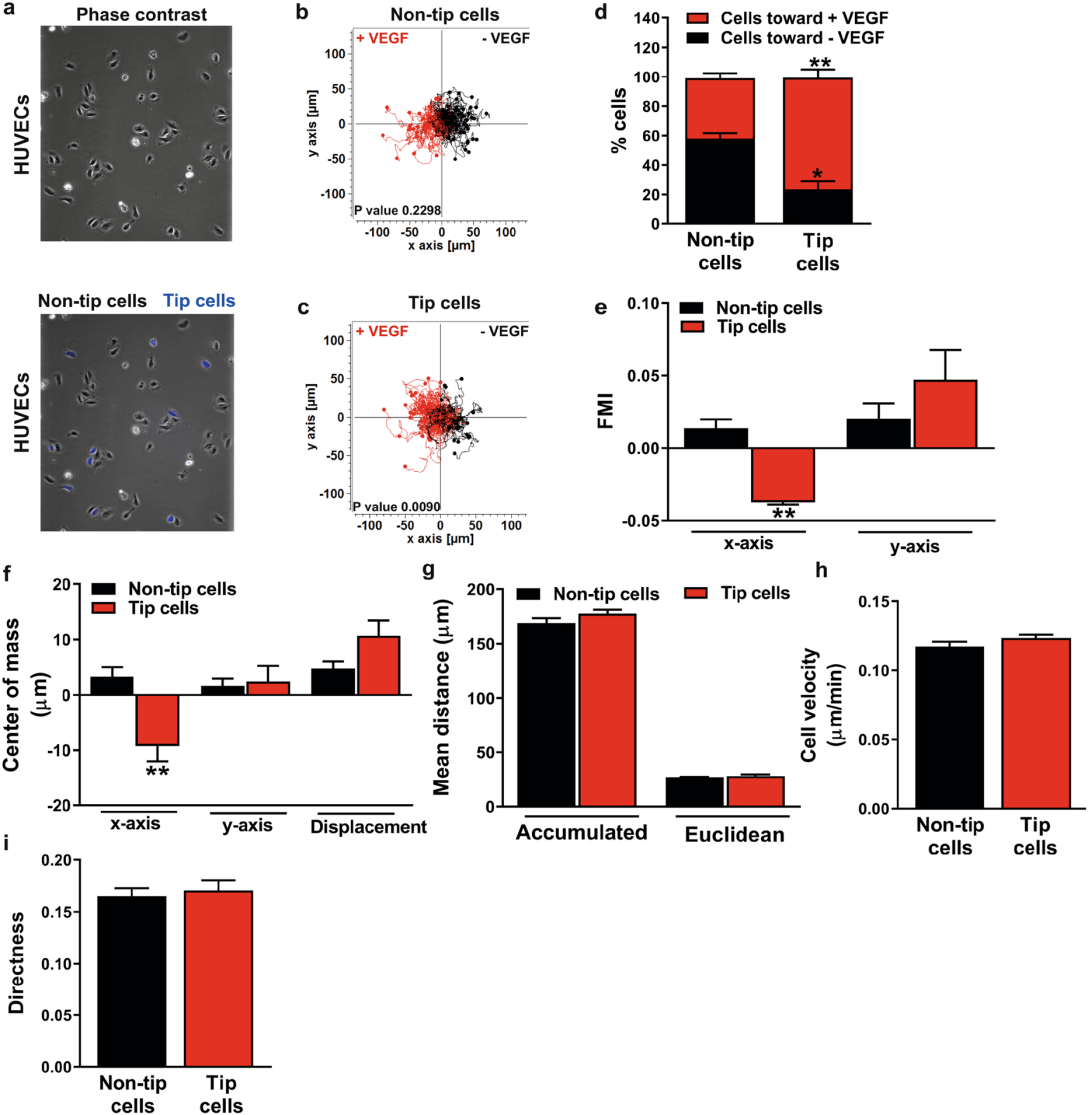
Migratory capacity of CD34^+^ tip cells and CD34^-^ non-tip cells toward VEGF. (**a**) FACS-sorted CD34^+^ tip cells were labelled using violet dye and mixed with unstained CD34^-^ non-tip cells and seeded in the observation area of the 2D µ-slide chemotaxis. Tracks of CD34^-^ non-tip cells (**b**) and CD34^+^ tip cells (**c**) are plotted in the 2D µ-slide chemotaxis for a duration of 24 h. Tracks of single cells moving toward VEGF (left) are shown in red and single cells moving toward site without chemoattractant (right) are shown in black. (**d**) The percentage of tip cells migrating toward VEGF was higher as compared to that of non-tip cells. (**e**) Tip cells migrate more perpendicular to VEGF (x-axis) as compared to non-tip cells, whereas the migration parallel to VEGF was similar in tip cells and non-tip cells. (**f)** The center of mass on the x-axis show that tip cells migrate more perpendicular to VEGF as compared to non-tip cells. No differences were found in accumulated distance and euclidean distance (**g**), velocity (**h**) and the directness (**i**) between tip cells and non-tip cells. FMI: forward migration index. Results are shown as ± SEM of experiments with HUVECs of at least 3 donors. *P < 0.05 and **P < 0.01 as compared to non-tip cells (Unpaired Student’s t-test). P-values in fig. (**b**,**c**) were given by the statistical Rayleigh test.

To study VEGF-directed migration of tip cells and non-tip cells, tip cells were separated from non-tip cells by FACS sorting of HUVECs with the use of anti-CD34 antibody. Isolated fractions of tip cells were labeled with CellTrace^TM^ violet and mixed with non-tip cells and cells were seeded in the observation area of the 2D µ-slide chemotaxis (Fig. 3a). The effect of VEGF on directed migration of tip cells and non-tip cells was investigated in a chemotaxis assay by tracking single cell movement for 24 h (Fig. 3b,c, respectively).

The percentage of tip cells that migrated toward VEGF was significantly higher as compared to that of non-tip cells (Fig. 3d). The forward migration index (FMI), represents the efficiency of forward migration of tip cells and non-tip cells parallel to VEGF (y-axis) and perpendicular to VEGF (x-axis), respectively. FMI x-axis values show that tip cells migrate more perpendicular to VEGF as compared to non-tip cells, whereas migration parallel to VEGF was similar in tip cells and non-tip cells (Fig. 3e). The center of mass (the spatial averaged point of all cell endpoints) on the x-axis demonstrate that tip cells migrate more perpendicular to VEGF as compared to non-tip cells. No differences were found in the center of mass on the y-axis (parallel to VEGF) and the displacement of the center of mass (differences in the center of mass at the beginning and end of the experiment) (Fig. 3f). In addition to attraction toward VEGF, single parameters of chemotactic migration were evaluated. No differences between tip cells and non-tip cells were found in mean accumulated distance and mean euclidean distance (the direct distance between starting point and endpoint of a cell track) (Fig. 3g), velocity (Fig. 3h) and the directness (straightness of cell trajectories) (Fig. 3i) .

These findings suggest that there are no differences in cellular movement itself (chemokinesis) in tip cells as compared to non-tip cells, but that VEGF is necessary for the directional migration of tip cells.

### Tip cells are less glycolytic than non-tip cells

**Figure 4:**
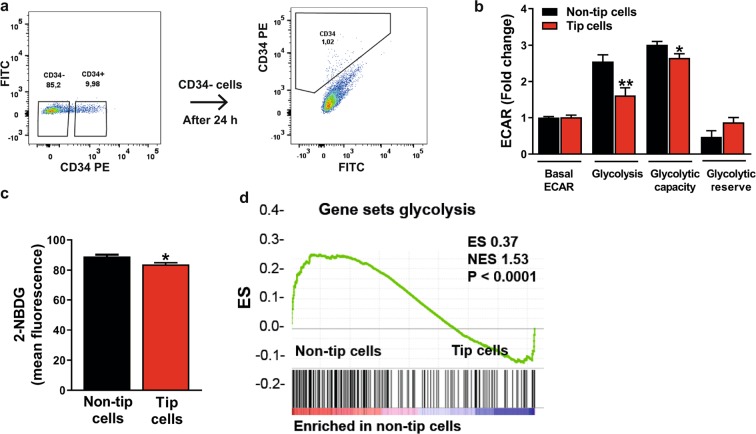
Glycolysis in tip cell and non-tip cell HUVECs. (**a**) FACS sorting of HUVECs with the use of PE-conjugated anti-CD34 antibody. Non-tip cells were put back into culture and their CD34 expression was checked after 24 h showing a percentage of tip cells of 1%. Assessment of glycolysis (ECAR) (**b**) and uptake levels of 2-NBDG (100 µM) (**c**) in CD34^-^ and CD34^+^ FACS-sorted HUVECs showed that the glycolysis rate was lower in tip cells. (**d**) GSEA metabolic pathway enrichment analysis showed an enrichment of the expression of glycolytic genes in non-tip cells as compared to tip cells. ECAR measurements are represented as fold change compared to basal ECAR levels in non-tip cells. mpH/min: milli-pH units per minute; ES: enrichment score; NES: normalized enrichment score. Results are shown as means ± SEM of experiments with HUVECs of at least 3 donors. *P < 0.05 and **P < 0.01 as compared to non-tip cells (Unpaired Student’s t-test).

ECs have a relatively high glycolytic activity, which is further increased during angiogenesis^11^. To study glycolysis in tip cells as compared to non-tip cells in more detail, tip cells were separated from non-tip cells by FACS sorting of HUVECs with the use of an anti-CD34 antibody. To check for purity, the non-tip cell fraction was reanalyzed after 24 h, and was shown to contain only 1% tip cells (Fig. 4a).

Glucose-induced glycolysis and glycolytic capacity was respectively 1.5-fold and 1.2-fold lower in tip cells as compared to non-tip cells (Fig. 4b), whereas glucose uptake was 6% lower in tip cells as compared to non-tip cells (Fig. 4c).

In line with these observations, gene set enrichment analysis (GSEA) performed on previously obtained microarray data of tip cells and non-tip cells^12^ (GSE 34850) showed an enrichment of gene sets for glycolysis in non-tip cells as compared to tip cells (Fig. 4d and supplementary Fig. S2).

Overall, these experiments show that in HUVEC cultures, tip cells are less glycolytic and take up lower amounts of glucose than non-tip cells.

### ECs have a flexible metabolism and mitochondrial respiration is essential for non-tip cell proliferation

**Figure 5:**
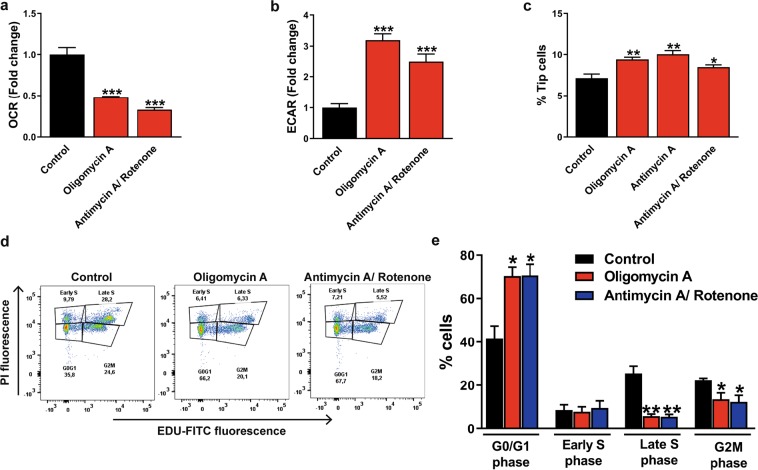
Effects of inhibition of mitochondrial respiration on glycolysis and HUVEC proliferation. Specific inhibitors of the oxidative phosphorylation, oligomycin A (1.5 µM) or antimycin A (2.5 µM)/rotenone (1.25 µM), reduced mitochondrial respiration (OCR) (**a**) and increased glycolysis (ECAR) (**b**) in HUVECs after 24 h of treatment. (**c**) Inhibition of the oxidative phosphorylation induced an increase in the percentage of tip cells. (**d**) Cell proliferation assay of HUVECs in the presence of oligomycin A or antimycin A/rotenone expressed as percentages of cells in the G0/G1, S, and G2M phase after FACS analysis of fluorescence of incorporated EdU. (**e**) Inhibition of the oxidative phosphorylation lowered percentages of HUVECs in the G2M phase and late S phase and elevated percentages of HUVECs in the G0/G1 phase. OCR and ECAR measurements were represented as fold change compared to control basal OCR and ECAR levels, respectively. Results are shown as means ± SEM of experiments with HUVECs of at least 3 donors. *P < 0.05, **P < 0.01, and ***P < 0.001 as compared to non-tip cells (Unpaired Student’s t-test).

Differentiated ECs have been suggested to be more glycolytic in response to angiogenic activation. In addition, it has been hypothesized that EC differentiation is induced by a switch to glycolysis^10,11^. However, the relative contribution of different metabolic pathways in tip cells and non-tip cells and the metabolic flexibility of ECs was not studied before. Therefore, we investigated whether inhibition of mitochondrial respiration stimulates glycolysis and affects differentiation of tip cells and non-tip cells *in vitro*. For this purpose, mitochondrial respiration was inhibited by specific inhibitors of the oxidative phosphorylation pathway, including oligomycin A (complex V inhibitor), antimycin A (complex III inhibitor), and rotenone (complex I inhibitor) for 24 h at concentrations that lower oxygen consumption rate (OCR), and then glycolysis was measured (Fig. 1)^21^. Oligomycin A reduced mitochondrial respiration by 2-fold and antimycin A/rotenone reduced mitochondrial respiration by 3-fold in HUVECs at 24 h after treatment (Fig. 5a). Blocking mitochondrial respiration increased glycolysis 3-fold after oligomycin A treatment and 2.7-fold after antimycin A/rotenone treatment as compared to control in HUVECs (Fig. 5b).

Next, the effect of increased glycolysis and reduced mitochondrial respiration on the differentiation of tip cells/non-tip cells was tested. Blocking mitochondrial respiration raised the percentage of tip cells (from 7.1% to 9.4% after oligomycin A, from 7.1% to 10% after antimycin A, and from 7.1% to 8.5% after antimycin A/rotenone treatment) at 24 h after treatment as compared to control (Fig. 5c).

To determine whether mitochondrial respiration is necessary for the proliferation of non-tip cells, the effect of oligomycin A and antimycin A/rotenone treatment on proliferation was measured after 24 h. Increased percentages of cells in the G0/G1 phase (from 40% to 70%) and decreased percentages of cells in the G2M phase (from 22% to 14%) and in the late S phase (EdU-positive cells) (from 25% to 6%) were found after blocking mitochondrial respiration as compared to control (Fig. 5e).

These findings indicate that blocking of mitochondrial respiration induces a switch to glycolysis, stimulates tip cell differentiation, and inhibits cell proliferation. This suggests that ECs have a flexible metabolism and that mitochondrial respiration is essential for non-tip cell proliferation.

### Tip cells have a higher mitochondrial respiration capacity

**Figure 6:**
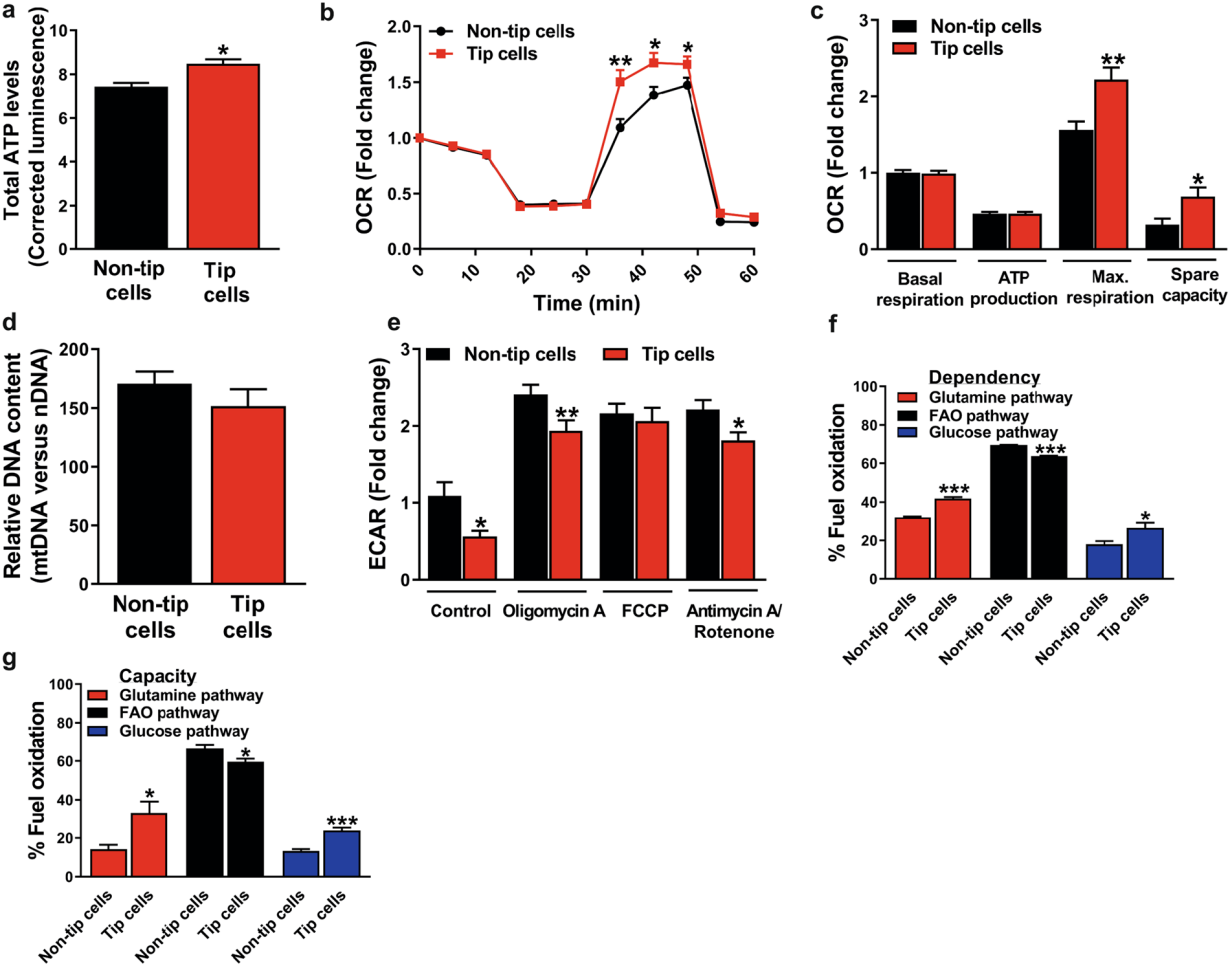
Mitochondrial respiration in tip cell and non-tip cell HUVECs. (**a**) Tip cells showed higher total ATP levels than non-tip cells (measured as relative luminescence). (**b**) Mitochondrial respiration in CD34^-^ and CD34^+^ FACS-sorted HUVECs. (**c**) Quantification of OCR in non-tip cells and tip cells revealed no difference in basal mitochondrial respiration and ATP production, but tip cells showed a higher maximum capacity and spare capacity of mitochondrial respiration. (**d**) Mitochondrial DNA revealed no differences between non-tip cells and tip cells. (**e**) Blocking mitochondrial respiration with oligomycin A (1.5 µM) and antimycin A (2.5 µM)/rotenone (1.25 µM) in tip cells and non-tip cells induced a switch to glycolysis, but to a lesser extent in tip cells. Mitochondrial fuel oxidation analysis showed the dependency (**f**) of tip cells and non-tip cells on the use of glucose, glutamine, and FAO, and their capacity (**g**) to oxidize these substrates in mitochondrial respiration. Tip cells showed a higher dependency on glucose and glutamine oxidation, whereas non-tip cells showed a higher dependency on FAO. Tip cells showed a higher capacity for glutamine and glucose oxidation, whereas non-tip cells showed a higher FAO capacity. OCR measurements were represented as fold change compared to basal OCR in non-tip cells. Luminescence was normalized for cell numbers. Results are shown as means ± SEM of experiments with HUVECs of at least 3 donors. *P < 0.05, **P < 0.01, and ***P < 0.001 as compared to non-tip cells (Unpaired Student’s t-test).

Since tip cells seem less glycolytic as compared to non-tip cells (Fig. 4), they may have a lower energy demand, or they may use other substrates than glucose for their energy demand and cell functioning. Therefore, we measured total ATP levels in tip cells and non-tip cells and found slightly higher ATP levels in tip cells (1.1-fold) as compared to non-tip cells (Fig. 6a).

Next, mitochondrial respiration was measured in tip cells and non-tip cells. Basal respiration and OCR linked to ATP production were similar, but maximal respiration capacity was 1.4-fold higher and spare respiratory capacity was 2.2-fold higher in tip cells as compared to non-tip cells (Fig. 6b,c). Similar results were found in tip cells in hMVEC cultures (Supplementary Fig. S3A). Mitochondrial DNA content in tip cells and non-tip cells was similar (Fig. 6d). Inhibition of mitochondrial respiration by oligomycin A and antimycin A/rotenone induced a switch to glycolysis in tip cells as well as in non-tip cells. However, the switch from mitochondrial respiration to glycolysis was smaller in tip cells as compared to non-tip cells. Uncoupling the mitochondrial respiratory chain and the oxidative phosphorylation with carbonyl cyanide 4-(trifluoromethoxy) phenylhydrazone (FCCP) also induced a switch to glycolysis, but did not show differences in glycolytic rate in tip cells and non-tip cells (Fig. 6e).

Together these results show that basal mitochondrial respiration and OCR linked to ATP production are similar in tip cells and non-tip cells. However, in conditions with increased energy demand, tip cells have a higher maximum capacity for mitochondrial respiration than non-tip cells. In addition, blocking mitochondrial respiration in tip cells and non-tip cells induces a switch to glycolysis, but to a lesser extent in tip cells.

### Tip cells are more flexible for substrate source with respect to their mitochondrial respiration

As our findings indicate that non-tip cells and tip cells both use mitochondrial respiration for their metabolism, we analyzed mitochondrial respiration in these subsets of cells in more detail. Various substrates can be used for mitochondrial respiration and ATP production in the presence of oxygen, such as fatty acids, glutamine, and glucose. Mitochondrial respiration of tip cells and non-tip cells was determined in the presence or absence of specific inhibitors of the respective pathways, i.e. UK5099 for inhibition of glucose oxidation, BPTES for inhibition of glutamine oxidation, and POCA for inhibition of fatty acid oxidation (FAO) (Fig. 1). Mitochondrial respiration measurements showed that both tip cells and non-tip cells are especially FAO dependent and have a high FAO capacity in comparison to that of glucose and glutamine (Fig. 6f,g). A higher dependency on glutamine oxidation and glucose oxidation was found in tip cells as compared to non-tip cells, whereas non-tip cells showed a relative higher FAO dependency (Fig. 6f). Additionally, tip cells showed a higher capacity for glutamine oxidation and glucose oxidation as compared to non-tip cells, whereas non-tip cells showed a relatively higher FAO capacity (Fig. 6g).

These data suggest that both tip cells and non-tip cells mainly use fatty acids as substrate for mitochondrial respiration but that tip cells, when compared to non-tip cells, are more dependent on glutamine and glucose oxidation for their mitochondrial functioning, but also have a higher capacity for glucose and glutamine oxidation when other fuel pathways are blocked.

### Regulation of relative tip cell numbers by TNF-a and VEGF treatment affects mitochondrial respiration and glycolysis

VEGF is a key regulator of angiogenesis and of tip and stalk cell differentiation^22,23^, whereas TNF-α is an anti-angiogenic regulator and reduces numbers of tip cells *in vitro* and i*n vivo*^12,24^.

**Figure 7:**
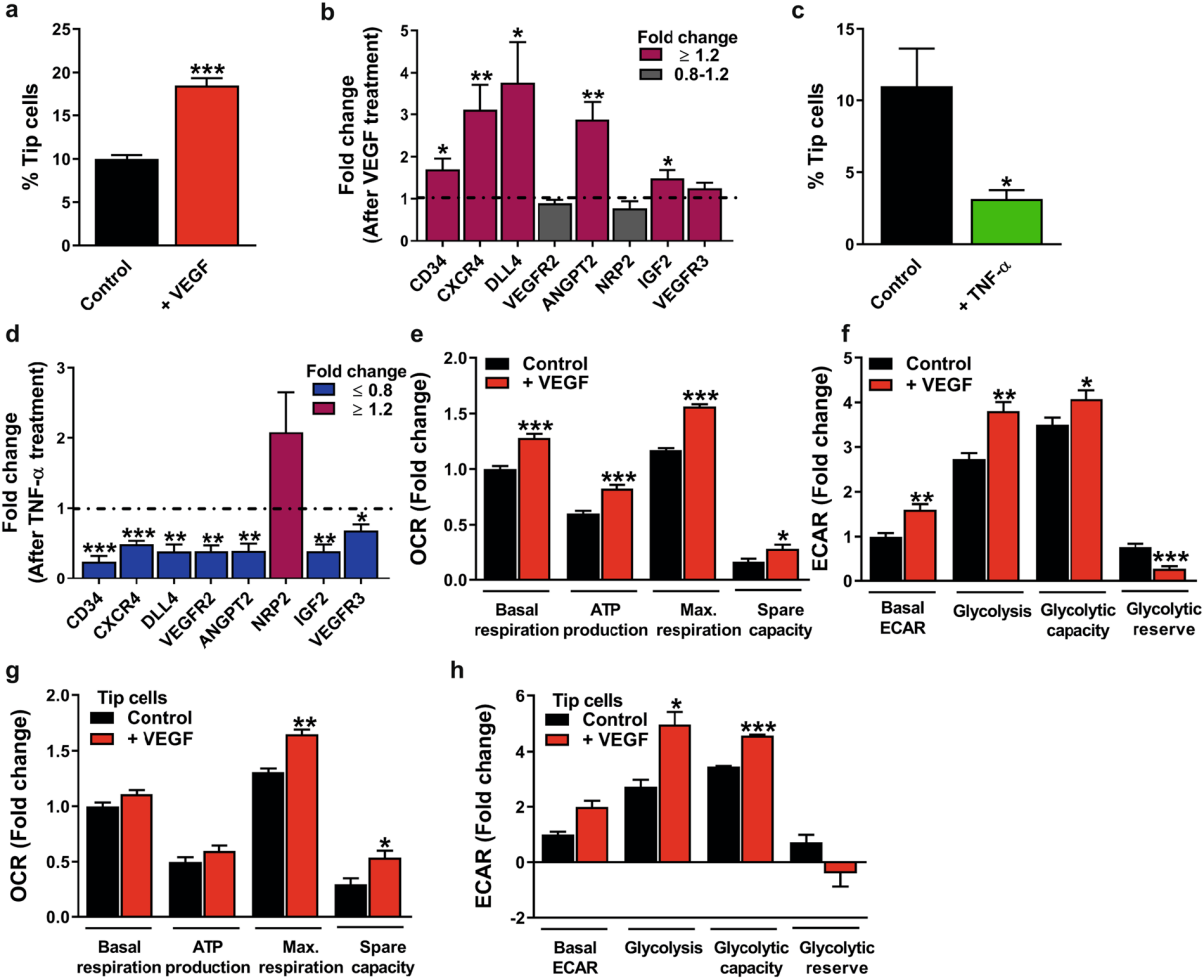
Effects of VEGF and TNF-α on tip cell differentiation and effects of VEGF on glycolysis and mitochondrial respiration. VEGF (25 ng/ml) treatment of HUVEC cultures increased relative tip cell numbers (**a**) and increased mRNA expression levels of 5 out of 8 tip cell-specific genes (**b**) at 24 h after treatment. Reduced percentage of tip cells (**c**) and reduced mRNA expression levels of 7 out of 8 tip cell-specific genes (**d**) were found in HUVEC cultures at 24 h after treatment with TNF-α (10 ng/ml). HUVEC cultures treated with VEGF showed induced mitochondrial respiration (**e**) and glycolysis (**f**). VEGF induced mitochondrial respiration (**g**) and glycolysis (**h**) in tip cells at 24 h after treatment. OCR and ECAR measurements were represented as fold change compared to control basal OCR and ECAR levels, respectively. Results are shown as means ± SEM of experiments with HUVECs of at least 3 donors. *P < 0.05, **P < 0.01, and ***P < 0.001 as compared to control (Unpaired Student’s t-test).

HUVECs and hMVECs were treated with VEGF or TNF-α for 24 h to determine whether differentiation of tip cells or non-tip cells affects glycolysis and/or mitochondrial respiration. The previously described induction and reduction of tip cell fractions of HUVECs after VEGF^22,23^ and TNF-α^12,24^ treatment, respectively, were confirmed (Fig. 7a,c) (similar results were found in hMVECs; Supplementary Fig. S3B,C). VEGF increased the fraction of tip cells from 10% to 18.5% (Fig. 7a), and increased the expression levels of 5 out of 8 tip cell-specific genes, including *CD34* (1.7-fold), *CXCR4*, *DLL4*, *ANGPT2* and *IGF2* (Fig. 7b). IGF2 was identified previously by our group as a tip cell-specific gene^12,16^. TNF-α reduced the percentage of tip cells from 11% to 3.2% (Fig. 7c), and decreased the expression levels of 7 out of 8 tip cell-specific genes, including *CD34* (4.1-fold) (Fig. 7d).

VEGF treatment of HUVEC cultures resulted in an induction of mitochondrial respiration (Fig. 7e) (confirmed in hMVECs; Supplementary Fig. S3D) as well as glycolysis (Fig. 7f) (reduced glycolytic reserve was found in hMVECs; Supplementary Fig. S3E). To determine the effects of VEGF treatment on glycolysis and mitochondrial respiration in tip cells and non-tip cells separately, extracellular acidification rate (ECAR), as a measure for glycolytic lactate production, and OCR, as a measure for mitochondrial respiration, were measured in both cell fractions after 24 h of VEGF treatment. In isolated populations of tip cells and non-tip cells, mitochondrial respiration (Fig. 7g and Supplementary Fig. S4A, respectively) and glycolysis (Fig. 7h and Supplementary Fig. S4B, respectively) were increased in both cell fractions after VEGF treatment. Tip cells may lose their tip cell phenotype when cultured in starvation medium after FACS sorting, and non-tip cells may be transformed into tip cells by VEGF. For this reason, we analyzed the percentage of *de novo* tip cell formation in isolated non-tip cell fractions after 24 h of culturing with VEGF. After treatment of non-tip cells with VEGF, the percentage of tip cells increased 2.5-fold as compared to cells in starvation medium (Supplementary Fig. S4C). Loss of tip cell phenotype is difficult to determine, since internalized CD34 is most likely still bound to anti-CD34 antibody after sorting and therefore not distinguishable from membrane-bound CD34. Therefore, we also examined the mRNA expression levels of CD34 and tip cell-specific genes in non-tip cells and tip cell cultures after 24 h VEGF treatment and found increased expression levels of 2 out of 8 tip cell-specific genes, *CXCR4* and *ANGPT2*, in both non-tip cells (Supplementary Fig. S4D) and tip cells (Supplementary Fig. S4E). These data indicate that non-tip cells can transform into the tip cell phenotype.

**Figure 8:**
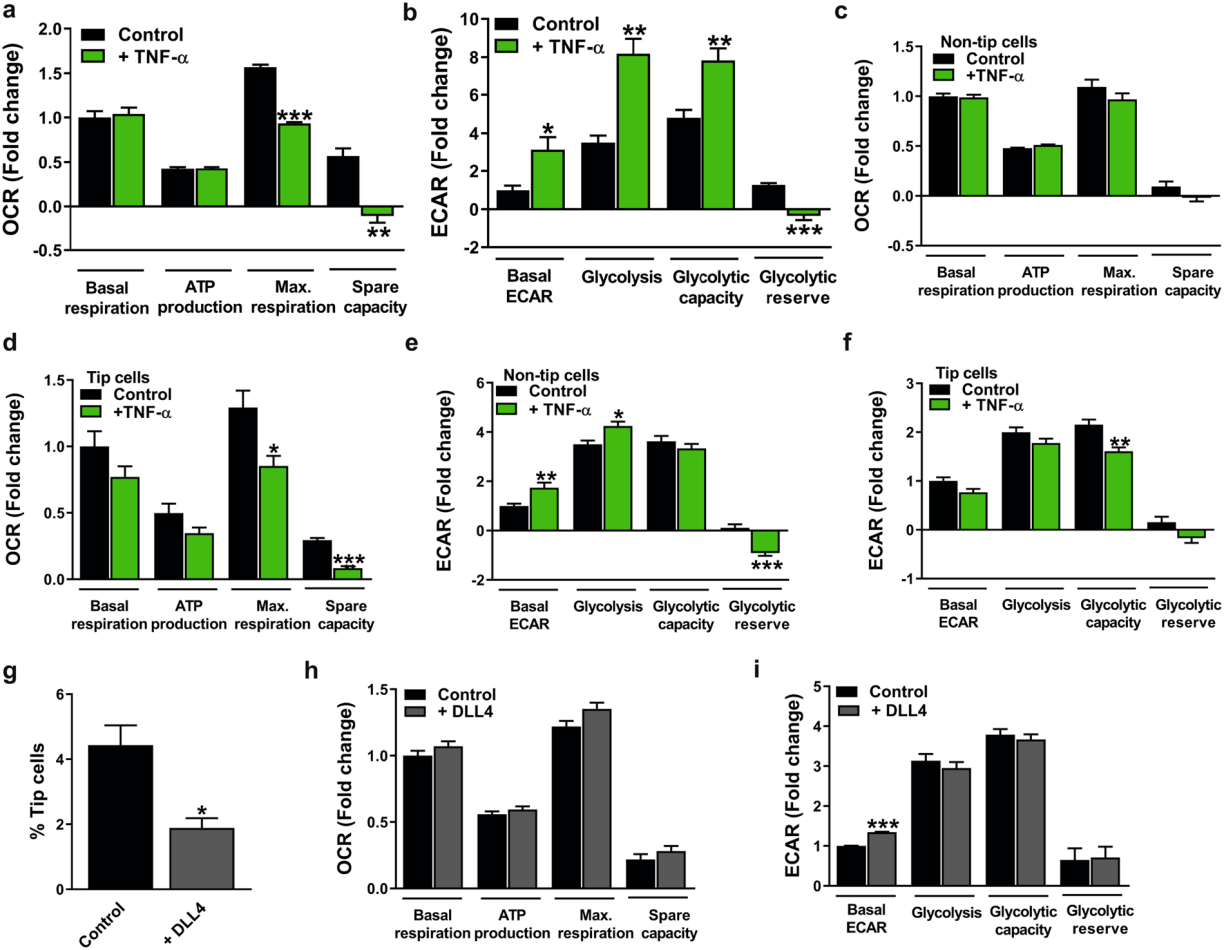
Effect of TNF-α in tip cells and non-tip cells and effect of DLL4 in total HUVEC populations on mitochondrial respiration and glycolysis. TNF-α (10 ng/ml) treatment of HUVEC cultures reduced maximal respiratory capacity and spare capacity (**a**) and induced glycolytic activity (**b**) at 24 h after treatment. TNF-α reduced maximal respiratory capacity and spare capacity in tip cells (**d**), whereas effects on mitochondrial respiration were not found in non-tip cells (**c**) at 24 h after treatment. (**e**) TNF-α in FACS-sorted HUVECs induced glycolysis in non-tip cells (**e**) and reduced glycolytic capacity in tip cells (**f**) at 24 h after treatment. (**g**) HUVECs cultured on DLL4-coated (1 µg/ml) plates showed lower tip cell percentages as compared to those cultured on BSA-coated (1 µg/ml) plates used as a control. DLL4 treatment did not affect mitochondrial respiration (**h**), but increased basal glycolysis (**i**) in HUVEC cultures at 24 h after cell addition. OCR and ECAR measurements were represented as fold change compared to control basal OCR and ECAR levels, respectively. Results are shown as means ± SEM of experiments with HUVECs of at least 3 donors. *P < 0.05, **P < 0.01, and ***P < 0.001 as compared to control (Unpaired Student’s t-test).

Next, the effects of TNF-α on mitochondrial respiration and glycolysis in HUVEC cultures were assessed after 24 h of incubation. TNF-α reduced the maximal respiratory capacity (1.7-fold compared to untreated control) and spare respiratory capacity (Fig. 8a) (confirmed in hMVECs; Supplementary Fig. S3F) and increased glycolysis by 2.3-fold (Fig. 8b) (reduced glycolysis found in hMVECs; Supplementary Fig. S3G) compared to control. After cell sorting, we found that TNF-α treatment of non-tip cells (Supplementary Fig. S5A) and tip cells (Supplementary Fig. S5B) resulted in decreased mRNA expression levels of tip cell-specific genes. This suggests that after TNF-α treatment, non-tip cells do not switch to tip cells and that tip cells lose their phenotype. Measurements of mitochondrial respiration and glycolysis in sorted cells after TNF-α treatment showed a reduction in maximal respiration capacity (1.5-fold compared to untreated control) and spare respiration capacity (3.5-fold compared to untreated control) exclusively in tip cells (Fig. 8c,d), and increased glycolysis in non-tip cells (Fig. 8e). Tip cells treated with TNF-α showed a decreased glycolytic capacity (1.3-fold compared to untreated control) (Fig. 8f).

Overall, these data indicate that induction of tip cell numbers by VEGF also increases mitochondrial respiration and glycolysis in total HUVEC cultures (tip cells and non-tip cells), and in tip cells in particular. TNF-α reduces maximal respiration capacity and spare capacity and induces glycolysis in total HUVEC cultures, whereas TNF-α treatment showed exclusively in tip cells reduced maximum capacity for mitochondrial respiration.

### Regulation of tip cell and non-tip cell numbers by Dll4-notch signaling increases glycolysis in HUVEC and hMVEC cultures

The DLL4-Notch signalling pathway is a feedback mechanism of tip cells that instructs neighbouring cells to remain stalk cells. This phenomenon is fundamental for the differentiation of endothelial cells into tip cells and stalk cells during the angiogenic sprouting process in physiological and pathological conditions^22,23^. HUVECs and hMVECs were cultured on DLL4-coated plates to promote the stalk cell phenotype and glycolysis and mitochondrial respiration were assessed. The previously described reduction in tip cell numbers after DLL4 treatment^12,16^ was confirmed in HUVECs (Fig. 8g) and hMVECS (Supplementary Fig. S6a), respectively.

Mitochondrial respiration was not affected after DLL4 treatment in HUVEC and hMVEC cultures (Fig. 8h and supplementary Fig. S6b, respectively). Basal glycolysis was 1.4-fold higher in DLL4-treated HUVECs, but glucose-induced glycolysis, glycolytic capacity, and glycolytic reserve were similar (Fig. 8i). Basal glycolysis, glucose-induced glycolysis, and glycolytic capacity were respectively 2.2-fold, 1.6-fold, and 1.4-fold higher in DLL4-treated hMVECs as compared to control (Supplementary Fig. S6c).

These data suggest that promoting the stalk cell phenotype by activating the DLL4-Notch signaling pathway increases glycolysis in HUVECs and hMVECs, but does not affect mitochondrial respiration.

## Discussion

In the present study, we demonstrate that tip cells and non-tip cells both use glycolysis and mitochondrial respiration for their energy production. However, tip cells appear to be less glycolytic and to have a higher capacity to respond to metabolic stress than non-tip cells in HUVEC cultures. Furthermore, we show that upon blocking mitochondrial respiration, ECs adapt their metabolism by increasing glycolysis, and that non-tip cells lose their proliferation activity.

Metabolism is the basis of any biological function. It is required to generate energy and building blocks, and to remove metabolic waste. Different cell types can process different types of fuel and can carry out various metabolic processes. Recent studies indicate that metabolic pathways are essential in regulating angiogenesis, involving a metabolic switch during angiogenesis^11^. Since tip cells, stalk cells, and phalanx cells have different characteristics and distinct functions, metabolism may differ between these specialized ECs. Unravelling differences in metabolic pathways between tip cells and non-tip cells, and better understanding of the regulatory roles of these pathways in different EC phenotypes during angiogenesis may provide support for the proposed targeting of specific metabolic pathways in anti-angiogenesis therapies. In the following sections we will discuss our findings in more detail.

### CD34 is a marker for tip cells *in vitro*

Although we intensively investigated the tip cell characteristics of CD34^+^ cells in monolayers of ECs^12,16^, we now confirm the existence of CD34-expressing tip cells in a sprouting model. The model more closely resembles blood vessels *in vivo* and forms a perfusable lumen. Our finding is in concordance with other studies that show expression of CD34 in sprouting models *in vitro*^25,26^. Previously we have reported that mRNA levels of DLL4 are 7-fold higher in CD34^+^ cells as compared to CD34^−^ cells. Recently, it was shown that cells that have high levels of DLL4 also have high levels of CD34^25^.

Extracellular VEGF gradients guide angiogenic sprouting by directing tip cell migration, whereas flattening of VEGF gradients leads to the inhibition of tip cell migration^22^. Genome-wide mRNA profiling analysis of CD34^+^ tip cells revealed enrichment of biological functions related to migration^12^. However, the migratory capacity toward VEGF in CD34^+^ tip cells and CD34^−^ non-tip cells separately was not studied before. We demonstrated that there are no differences in cellular movement itself in tip cells and non-tip cells, but VEGF is necessary for the directional migration of tip cells. Taken together, our findings further verify that CD34 is a useful marker for tip cells *in vitro*.

### Reduced glycolysis in tip cells

Glycolysis is the metabolic pathway that converts glucose into pyruvate. Oxygen availability is one of the most important factors that determines the fate of pyruvate besides biosynthetic requirements (Fig. 1). In anaerobic conditions, pyruvate is converted to lactate by LDH-A, whereas in the presence of oxygen, pyruvate is oxidized in the mitochondria unless biosynthesis requires aerobic glycolysis^27^. Previous work has suggested that ECs rely mainly on glycolysis for ATP production, and that upon activation during angiogenesis glycolysis is further enhanced^11^. Elevated activity of the key glycolytic enzyme PFKFB3 was shown to promote a migratory tip cell phenotype, suggesting that tip cell formation is driven by glycolysis^11^, and that non-proliferating quiescent ECs are less glycolytic as compared to angiogenic ECs^10^. Our findings are a further specification of this notion, since we were able to do our investigations on separated tip and non-tip cell fractions. We observed that in EC cultures, tip cells are relatively less glycolytic than non-tip cells and switch to glycolysis to a lesser extent when mitochondrial respiration is inhibited. In both spheroid assays *in vitro*, and in vessel sprouting and outgrowth *in vivo*^11^ induced by activation of the Notch signaling pathway by DLL4, others have shown that inhibition of PFKFB3 in ECs impairs both glycolysis and vessel sprouting^28^. Overexpression of PFKFB3 overcomes this DLL4-Notch-induced suppression of EC proliferation and glycolysis^28^. Because tip cells do not proliferate and stalk cells do during angiogenesis, we conclude that these Notch effects on glycolysis and proliferation occur only in stalk cells. However, our findings that DLL4 increases glycolysis in ECs are in contrast with the findings by others that Notch signaling downregulates glycolysis^11,28^. This controversy needs to be elucidated but we like to note that DLL4-Notch signaling affects proliferating stalk cells *in vivo* and thus upregulation of glycolysis and EC proliferation by DLL4-Notch signaling fits better in the concept of non-proliferating tip cells and proliferating stalk cells.

### Mitochondrial respiration is necessary for EC proliferation

Although previous studies suggest that ECs rely mainly on glycolysis, they have the ability to use mitochondrial respiration since ECs line blood vessels and have therefore immediate access to oxygen. We demonstrated that mitochondrial DNA content is identical in tip cells and non-tip cells *in vitro*. In addition to glucose, ECs are well-equipped to metabolize exclusively mitochondrially-oxidizable substrates such as glutamine and fatty acid^29,30^. Activity of glutaminase, an enzyme that catalyzes the conversion of glutamine into glutamate, has been reported to be high in ECs^31^. Glutamine contributes to ATP production and cell survival in ECs, when glucose supply is decreased^32^. FAO is crucial for biosynthesis, to generate nucleotides for DNA replication during cell proliferation. Additionally, FAO is involved in angiogenesis because FAO stimulates vessel sprouting via EC proliferation and because FAO activity is increased during glucose deprivation^33,34^. Consistent with earlier findings^35,36^, we showed that upon blocking of mitochondrial respiration, HUVEC proliferation is inhibited, even when glycolysis is increased, an effect most likely occurring in non-tip cells, as tip cells do not proliferate. This suggests that, although constitutive glycolysis is higher in non-tip cells, this fraction is dependent on mitochondrial respiration for proliferation. Mitochondria have been reported for their bioenergetic properties. However, mitochondrial reactive oxygen species (ROS) production is known to be involved in a wide range of cellular processes, including cell proliferation^37^. Taken together, it may be possible that non-tip cells rely on mitochondrial respiration for their proliferation, but derive their ATP mainly from glycolysis.

We also observed that the tip cell fraction *in vitro* was increased after blocking mitochondrial respiration, suggesting that an experimentally-induced increase in glycolysis stimulates differentiation into tip cells. This is in line with *in vivo* experiments reported in the literature in which ECs with experimentally-induced higher glycolysis took the tip cell position and in which cells with experimentally-induced lower glycolysis favored the stalk cell position^11^. Because we observed lower constitutive glycolysis in the tip cell fraction, our results indicate that metabolic pathways involved in induction of the tip cell phenotype are probably not related to the constitutive metabolic requirements of tip cells.

### ECs can adapt their metabolism to microenvironmental conditions

Hypoxia induces angiogenesis, but is also known as a biologically unbalanced status that is referred to as cycling hypoxia^31,38^. Angiogenesis is triggered under hypoxic conditions, which initiates tip cells to migrate into the extracellular matrix toward the hypoxic area. Migrating tip cells and proliferating stalk cells are both exposed to hypoxia during angiogenesis and are oxygenated only when a new vascular loop is established by connecting two sprouts of endothelial tip cells, referred to as a perfused neovessel^39^. Adaptation to oxygenated or hypoxic environments requires from the ECs involved to have a metabolism that can meet such oxygen fluctuations. Here, we demonstrate that HUVECs can switch to glycolysis when mitochondrial respiration is blocked, showing that HUVECs are not dependent on a specific metabolic pathway, but that they can adapt their metabolism on microenvironmental circumstances. Furthermore, we showed that tip cells are less glycolytic and have a higher maximal respiration capacity and spare respiratory capacity as compared to non-tip cell HUVECs.

No differences were found in basal mitochondrial respiration and OCR linked to ATP production between tip cells and non-tip cell HUVECs. Additionally, TNF-α treatment, which acts as an anti-angiogenic signal that reduces the number of tip cells *in vitro* and *in vivo*^12^, induced glycolysis and reduced mitochondrial respiration in HUVEC cultures. Although we need to be careful with our interpretations, since TNF-α also has pro-inflammatory effects, we did find a specific downregulation of mRNA levels of tip cell genes, suggesting a specific decrease in the percentage of tip cells. We demonstrated that maximal respiration capacity and spare capacity are exclusively decreased in tip cells as compared to non-tip cells after TNF-α treatment. This indicates that tip cells are less dependent on glycolysis for their functioning, and that under basal conditions, tip cells and non-tip cells both use mitochondrial respiration for their energy demand and/or cell functioning. However, in conditions of increased energy demand, tip cells show a higher maximum capacity of mitochondrial respiration and a higher ability to respond to an increased energy demand as compared to non-tip cells. Additionally, tip cells demonstrate a higher dependency on glutamine oxidation and glucose oxidation, and also have a higher capacity for glucose oxidation and glutamine oxidation as compared to non-tip cells. We also showed that both tip cells and non-tip cells use high rates of FAO for their mitochondrial functioning, when compared to glucose oxidation and glutamine oxidation. This suggests that fatty acids are an important substrate for mitochondrial functioning of tip cells and non-tip cells, and that tip cells can rely more on glutamine and glucose oxidation as well, showing that tip cells are more flexible in their substrate source. The reliance of tip cells on glutamine oxidation support recent studies that show that glutamine oxidation is essential for ECs to reach the tip position in vessel sprouts. In addition, glutamine deprivation reduces EC proliferation (most likely occurring in non-tip cells)^40^. This suggests that tip cells use glutamine as a substrate for their migratory behavior, whereas non-tip cells use glutamine for their proliferative behavior.

### VEGF regulates tip cell differentiation and affects glycolysis and mitochondrial respiration

VEGF is an important regulator of blood vessel formation and function^22^. It controls several processes in angiogenic sprouting, including migration, EC proliferation and survival, and induces the differentiation into tip cells or stalk cells via binding to VEGFR2^41^. When quiescent ECs are activated by VEGF during angiogenesis, glycolysis is induced by upregulating the expression of glucose transporter 1 (GLUT-1), PFKFB3 and LDH-A^11,42,43^. Since VEGF acts as a pro-angiogenic growth factor, the ability of VEGF to regulate glucose transport in association with EC differentiation may serve to ensure successful angiogenesis. We show here that VEGF increases percentages of tip cells in HUVEC populations. In addition, gene expression of tip cell markers was increased in HUVEC populations as well as in populations of isolated tip cells after VEGF treatment. Tip cell markers are not exclusively expressed in tip cells, but are expressed at much higher levels in tip cells as compared to non-tip cells^12^. We observed a higher relative increase in expression of tip cell markers in unsorted HUVEC populations than in populations of tip cells after VEGF treatment. This is most likely caused by the larger tip cell fraction after VEGF treatment of HUVEC populations. In addition, expression of tip cell markers may be induced by VEGF in non-tip cells as well. In addition, VEGF stimulates mitochondrial respiration as well as glycolysis *in vitro*. Consistent with our findings, Sun *et al*.^44^ demonstrated that VEGF stimulates mitochondrial respiration. They showed that VEGF upregulates the mitochondrial transcription factor peroxisome proliferator-activated receptor gamma co-activator-1 alpha (PGC1α), a master regulator of mitochondrial biogenesis and oxidative metabolism^45^, and induces OCR. This indicates that, in addition to glycolysis, VEGF also stimulates and regulates mitochondrial respiration.

Consistent with the findings of Jakobsson *et al*.^46^, we demonstrated that, upon treatment with VEGF, non-tip cells transform into the tip cell phenotype. In addition, we found that tip cells do not change back to the non-tip cell phenotype when cultured in the presence of VEGF. Tip cells stimulated with VEGF showed increased glycolysis and mitochondrial respiration compared to unstimulated cells. This again suggests that VEGF is an important co-regulator of glycolysis and mitochondrial respiration in tip cells. Since VEGF induced the tip cell phenotype in non-tip cells, no conclusion could be drawn specifically on the effect of VEGF on glycolysis and mitochondrial respiration in sorted populations of non-tip cells.

In conclusion, our results show that ECs have a flexible metabolic phenotype, and suggest that a balance between several metabolic pathways is necessary for tip cells and non-tip cells to maintain their phenotype and for their proper functioning in angiogenesis. We have shown that tip cells need glycolysis to maintain their phenotype, but they use mitochondrial respiration in circumstances of high energy demand to respond to microenvironmental changes during angiogenesis. On the other hand, we demonstrated that non-tip cells are more glycolytic and that they need mitochondrial respiration for cell proliferation. Collectively, we demonstrated that in angiogenesis, glycolysis and mitochondrial respiration are tightly coupled and serve as a molecular interconversion in EC differentiation. Our studies were confined to an *in vitro* approach. CD34^+^ cells *in vitro* share almost all characteristics of tip cells *in vivo* and can therefore enable understanding of differentiation and functional regulation of tip cells versus non-tip cells. However, in this model, the complex and variable microenvironment of angiogenesis *in vivo* in neovascular eye diseases and tumors is missing. Complementary and confirmatory data from studies *in vivo* are necessary to completely understand molecular processes during angiogenesis^47^.

Recent studies in mouse models have shown the anti-angiogenic potential of metabolic manipulation in pathological angiogenesis *in vivo*^11,40,48^. In addition, combined inhibition of PFKFB3 and VEGF was shown to normalize the deregulated EC rearrangement as observed in pathological angiogenesis in mice *in vivo*^49^. Therefore, it was suggested that a combination of current anti-angiogenic therapies, such as anti-VEGF treatment, and metabolic targeting may have a higher therapeutic efficacy and specificity in human patients with cancer than current anti-angiogenic therapies. However, it is important to note that in the past, results of studies in tumor angiogenesis in mice have often failed to be representative for human tumor angiogenesis^50^. This may underlie the apparent discrepancies between the results of previous mouse studies and our findings in a model of human tip cells *in vitro*. The dynamic and complex metabolic networks in human tip cells that we report here challenge the suggested approaches to therapeutically target a single component of metabolic pathways as anti-angiogenic therapy. Our results suggest that such inhibition of EC energy metabolism in angiogenesis may activate compensatory pathways, which could still maintain EC differentiation and angiogenic sprouting.

## Material and Methods

### Cell cultures

Primary HUVECs were isolated from umbilical cords, as described earlier^12,51^, and grown in M199 basal medium (Gibco, Grand Island, NY, USA) supplemented with 10% heat inactivated human serum, 10% fetal bovine serum (Gibco), and 1% penicillin-streptomycin-glutamine (Gibco). hMVECs, a kind gift of Dr. P. Koolwijk (Amsterdam UMC, VU University Medical Center, Amsterdam, The Netherlands), were cultured with 50% HUVEC medium and 50% EBM-2 medium (Lonza, Basel, Switzerland) and cells were characterized as previously described^52^. HUVECs and hMVECs were cultured in 2% gelatin-coated (Millipore, Billerica, MA, USA) T75 culture flasks at 37 °C and 5% CO_2_. Experiments were performed with subconfluent HUVECs at passage 3–4 and hMVECs at passage 9–10 of at least 3 different donors. Human serum and umbilical cords were collected anonymously according to the principles of conduct for research integrity as described in the Research Code AMC VUmc, §3.2.

### Identification and isolation of tip cells

For determining the percentage of tip cells in cell cultures, cells were fixed in 4% paraformaldehyde in PBS for 10 min and incubated with anti-CD34-phycoerythrin (1:50; anti-CD34-PE; clone QBend-10, Thermo Scientific, Waltham, MA, USA) for 30 min at room temperature. Tip cells were identified as CD34-positive cells using a FACSCalibur (Beckton Dickinson, Franklin Lakes, NJ, USA) and analyzed using FlowJo 6.4.7 software (Tree Star, San Carlos, CA, USA). The FITC channel was used to detect autofluorescence. Non-stained and non-treated cells were used as negative controls. For cell sorting experiments, cells were sorted on the basis of CD34 expression (range between 5–20%) with anti-CD34-PE on a Sony SH800Z cell sorter (Sony Biotechnology, Tokyo, Japan). After cell sorting, CD34^−^ hMVECs and HUVECs were put back in culture for respectively 6 or 24 h, cells were fixed, stained and analyzed using flow cytometry as described above.

### Compounds/treatments

When indicated, cells were treated with VEGF_165_ (25 ng/ml; R&D Systems, Minneapolis, MN, USA) or TNF-α (10 ng/ml; ProSpec-Tany Technogene, Rehovot, Israel) in M199 medium supplemented with 2% human serum (starvation medium) for 24 h. Culture plates were coated with 0.2% gelatin in PBS containing bovine serum albumin (BSA) (1 µg/ml; Sigma-Aldrich, St. Louis, MO, USA) or DLL4 (1 µg/ml; R&D Systems) at 24 h prior to cell addition. Cells were treated with 2-deoxyglucose (2-DG) (100 mM; Sigma-Aldrich) for 0.5, 1, 4, and 24 h and with oligomycin A (1.5 µM; Cayman Chemical, Ann Arbor, MI, USA), antimycin A (2.5 µM; Sigma-Aldrich), and rotenone (1.25 µM; Sigma-Aldrich) for 24 h in HUVEC medium. A schematic overview of the relevant metabolic compounds is shown in Fig. 1.

### 3D angiogenic sprouting model

A 3D angiogenesis model was used as described previously^20^. Briefly, collagen type I (R&D systems) was patterned in the microfluidic channel, followed by a 24 h coating with fibronectin (10 µg/ml; Sigma-Aldrich) solution in PBS at 37 °C and 5% CO_2_. HUVECs were seeded in one of the adjacent channels in a concentration of 1·10^7^ cells/ml in EGM2 medium. The cells were cultured for 4 days to form a confluent microvessel. Sprouting was induced by supplementing EGM2 media with VEGF (50 ng/ml; Peprotech, Rocky Hill, New Jersey), S1P (1 µM; Sigma) and PMA (2 ng/ml; Sigma). The sprouting microvessels were fixed using 4% PFA for 15 minutes, permeabilized for 15 minutes using 0.2% Triton-X100 and stained for nuclei using Hoechst (1:2000), F-actin using phalloidin (1:200) and anti-CD34 (1:50; clone MD34.2, Sanquin, Amsterdam, The Netherlands) at room temperature. Images were acquired using a confocal microscope (ImageXpress Micro, Molecular Devices) using a 20X Super Plan Fluor objective (NA 0.45) and images were acquired in the DAPI, FITC and TRITC channels. Image depth (bits) was set at 16, binning at 2, imaging resolution at 1024 × 1024 (0.702 µm/pixel). 80 Z-steps were acquired with 1 µm distance per site, and a total of 4 sites with 10% overlap were acquired per well. The max projections were stitched in FIJI using the pairwise stitching plugin with fusion method ‘linear blending’^53^.

### 2D µ-slide chemotaxis

The migration capacity of tip cells and non-tip cells was determined using the 2D µ-slide chemotaxis (Ibidi GmbH, Munich, Germany), according to the manufacturer’s protocol. Briefly, HUVECs were sorted on the basis of CD34 expression with anti-CD34-PE (Thermo Scientific) on a Sony SH800Z cell sorter (Sony Biotechnology) and CD34^+^ HUVECs were labeled with CellTrace^TM^ violet dye (Thermo Scientific), according to the manufacturer’s protocol. After cell labeling, CD34^+^ cells were mixed with unstained CD34^−^ cells and mixed cells were seeded in the observation area of the 2D µ-slide chemotaxis. After cell adhesion, VEGF_165_ (25 ng/ml; R&D Systems) supplemented in HUVEC medium was added to the left reservoir of the 2D µ-slide chemotaxis chamber and HUVEC medium without additional VEGF was added to the right reservoir of the chamber. The 2D µ-slide chemotaxis was incubated at 37 °C in an atmosphere containing 5% CO_2_ under an inverted microscope (Leica; 10x objective) and the effect of VEGF-directed migration of tip cells and non-tip cells was investigated by tracking single cell movement. Live cell images were acquired every 10 min for 24 h at 3 fixed spots per chamber and time lapse movies were analyzed by manual tracking of single cell movement using ImageJ. The cells that were lost due to cell proliferation, cell death, or cells leaving the edges of the observation area were excluded from statistical analysis. For plotting and analyzing the tracked data, the software tool provided by the manufacturer was used. A detailed description of all parameters of the 2D µ-slide chemotaxis was published by Zengel *et al*.^54^.

### Measurement of cellular metabolism: flux analysis

OCR, a measure of oxygen utilization, of cells is an important indicator of mitochondrial function^21^. ECAR is a measure of lactic acid levels, formed during the conversion of glucose to lactate during glycolysis^55^. OCR and ECAR were measured using Seahorse XF96 extracellular flux analyzer (Seahorse Bioscience Europe, Copenhagen, Denmark). HUVECs and hMVECs were seeded at 40,000 cells per well in Seahorse XF96 polystyrene tissue culture plates (Seahorse Bioscience Europe) and incubated overnight. Prior to measurement, cells were incubated in unbuffered DMEM assay medium (Sigma-Aldrich) in a non-CO_2_ incubator at 37 °C for 1 h. Both OCR and ECAR were measured every 4 min with a mixing of 2 min in each cycle, with 4 cycles in total. Compounds for OCR measurements were added to DMEM assay medium (Sigma-Aldrich) containing glucose (25 mM; Sigma-Aldrich), sodium pyruvate (1 mM; Gibco), and glutamine (2 mM; Gibco) and were used at the following concentrations: oligomycin A (1.5 µM), FCCP (1.5 µM; Sigma-Aldrich), antimycin A (2.5 µM; Sigma-Aldrich) and rotenone (1.25 µM; Sigma-Aldrich). This allowed for calculation of OCR linked to ATP production, maximal respiration capacity and spare respiratory capacity. Basal respiration was measured prior to injection of oligomycin A. Compounds for ECAR measurements were added to DMEM assay medium (Sigma-Aldrich) containing glutamine (2 mM; Gibco) and were used at the following concentrations: glucose (10 mM; Sigma-Aldrich), oligomycin A (1.5 µM), and 2-DG (100 mM; Sigma-Aldrich). This allowed for calculation of glycolysis rate, glycolytic capacity, and glycolytic reserve. Basal ECAR was measured prior to injection of glucose. The Seahorse XF Mito fuel flex test was used to test the cells’ ability to switch oxidative pathways in meeting basal energetic demands and provides information regarding the relative contributions of glucose, glutamine and long chain fatty acid oxidation to basal respiration. The following compounds were used: 2-cyano-3-(1-phenyl-1H-indol-3-yl)-2-propenoic acid (UK5099) (2 µM; Sigma-Aldrich), bis-2-2(5-phenylacetamido-1,3,4-thiadiazol-2-yl) ethyl sulfide (BPTES) (3 µM; Sigma-Aldrich), sodium 2-[5-(4-chlorophenyl)pentyl]-oxirane-2-carboxylate (POCA) (100 µM) for inhibition of the glucose, glutamine, and fatty acids oxidation pathway, respectively. Dependency of cells on a particular fuel pathway was analyzed after inhibiting the pathway of interest followed by the two alternative pathways and measurement of OCR. The mitochondrial capacity (cells ability to oxidize a fuel when other fuel pathways are inhibited) was measured by inhibiting the two alternative pathways followed by the pathway of interest. To correct for differences in cell numbers, DNA content was determined using CyQUANT Cell Proliferation Assay (Thermo Scientific) and a microplate reader (BMG LABTECH; CLARIOstar, Ortenberg, Germany) after each experiment, according to the manufacturer’s instructions. Each measurement is the average of triplicate wells and is represented as fold change compared to basal OCR or ECAR. A schematic overview of glycolysis and mitochondrial respiration and the used compounds is shown in Fig. 1.

### *In vitro* glucose uptake

HUVECs were sorted with the use of anti-CD34 antibody and grown overnight in culture plates. After 24 h, cells were incubated with a fluorescent D-glucose analog 2-[N-(7-nitobenz-2-oxa-1,3-diazol-4-yl)-amino]-2-deoxy-D glucose (2-NBDG) (100 µM; Thermo Scientific) in HUVEC medium for 1 h at 37 °C. 2-NBDG fluorescence was measured in the FITC channel using a FACSCalibur (Beckton Dickinson) and analyzed using FlowJo 6.4.7 software (Tree Star).

### ATP measurement

Total ATP levels (intracellular and extracellular ATP levels) were measured using Cell titer Glo luminescent cell viability assay kit (Promega, Madison, WI, USA) according to the manufacturer’s protocol. The luminescent signal was measured using a microplate reader (BMG LABTECH; CLARIOstar) and normalized to the number of cells and the background signal from serum-supplemented medium without cells. All experiments were performed in triplicate.

### Cell viability assay

A 3-(4,5-dimethylthiazol-2-zyl)-2,5-dipenyl-tetra-zolium bromide (MTT) assay (Promega) was used to test the viability of cells after 2-DG treatment (after 0.5, 1, 4, and 24 h), according to the manufacturer’s instructions. Absorbance was photometrically measured at 570 nm using a microplate reader (VersaMax Microplate reader, Sunnyvale, CA, USA).

### RNA isolation, cDNA synthesis, and quantitative PCR

Total RNA was isolated using TRIzol (Invitrogen, Carlsbad, CA, USA) at 24 h after treatment, according to the manufacturer’s instructions. Briefly, total RNA was measured on a nanodrop (ND-100; NanoDrop Technologies, Wilmington, DE, USA). RNA (1 µg) was DNase-I (amplification grade; Invitrogen) treated and reverse transcribed into first strand cDNA using the Maxima first strand cDNA synthesis kit (Thermo Scientific). Real-time quantitative PCR was performed on 20x diluted cDNA samples using a CFX96 system (Bio-Rad, Hercules, CA) and specificity of primers was confirmed as described previously^56^. Primers are listed in Supplementary Table 1. Ct values were converted to arbitrary absolute amounts (2^−Ct^ × 1E12) and expressed as fold change as compared to controls. Gene expression data was normalized to tyrosine 3-monooxygenase/tryptophan 5-monooxygenase activation protein and zeta polypeptide (YHWAZ), as determined by NormFinder^57^. Each measurement was performed in duplicate and was presented as fold change as compared to control.

### Cell cycle analysis

Cell proliferation was assessed using Click-iT^TM^ Plus EdU flow Cytometry Assay Kit (Thermo Scientific), according to the manufacturer’s instructions. Briefly, 10 µM 5-ethynyl-2’-deoxyuridine (EDU) was added to adherent subconfluent HUVEC cultures and cells were incubated in HUVEC medium for 24 h at 37°C. Cell cycle analysis was determined by flow cytometry with 488 nm excitation (Beckton Dickinson).

### Genomic DNA isolation

Prior to genomic DNA isolation, HUVECs were FACS sorted with the use of anti-CD34-PE antibody, as described above, and total DNA was extracted using QIAamp DNA mini kit (QIAGEN), according to the manufacturer’s instructions. Total DNA was measured on a NanoDrop (ND-100; NanoDrop Technologies) and samples were normalized to 1 ng/µl genomic DNA. Genomic DNA was amplified using qPCR as described above. Primers to determine mitochondrial DNA and nuclear DNA are listed in Supplementary Table 2.

### Gene set enrichment analysis

Changes in the expression of functionally-related genes at the genome-wide expression profile level were detected using gene set enrichment analysis (version 2.2.3; Broad Institute, Cambridge, MA, USA). The following gene sets were used: Hallmarks gene set (h.all.v5.2.symbols.gmt); Gene Ontology gene set (c5.all.v5.2.symbols.gmt). The number of permutations was set at 100 and a false discovery rate (FDR) Q value < 25% was used as criterion for significantly enriched gene sets.

### Statistical analysis

All experiments were performed in ECs of at least 3 donors and were performed in duplicate or triplicate. All data were expressed as mean ± standard error of the mean (SEM). GraphPad Prism 6 software was used to asses statistical significance by a two-tailed Student’s t-test. Statistical significance was defined as * p < 0.05, ** p < 0.01, *** p < 0.001. To correct for differences between donors, factor correction, as described previously^58^, was used for flow cytometry data and Seahorse flux data.

## Supplementary information

Supplementary info

## Data Availability

The raw datasets generated during and/or analyzed during the current study will be made available upon request to the corresponding author.

## References

[1] Witmer AN, Vrensen GF, Van Noorden CJ, Schlingemann RO (2003). Vascular endothelial growth factors and angiogenesis in eye disease. Progr. Ret. Eye Res..

[2] Kumar N (2013). Retinal pigment epithelial cell loss assessed by fundus autofluorescence imaging in neovascular age-related macular degeneration. Ophthalmology.

[3] Sankar MJ, Sankar J, Mehta M, Bhat V, Srinivasan R (2016). Anti-vascular endothelial growth factor (VEGF) drugs for treatment of retinopathy of prematurity. Cochrane Database Syst. Rev..

[4] Folkman J, Klagsbrun M (1987). Angiogenic factors. Science.

[5] Hanahan D, Weinberg RA (2011). Hallmarks of Cancer: The Next Generation. Cell.

[6] Verheul HM, Voest EE, Schlingemann RO (2004). Are tumours angiogenesis-dependent?. J. Pathol..

[7] Geudens I, Gerhardt H (2011). Coordinating cell behaviour during blood vessel formation. Development.

[8] Siemerink MJ, Klaassen I, Van Noorden CJ, Schlingemann RO (2013). Endothelial tip cells in ocular angiogenesis: potential target for anti-angiogenesis therapy. J Histochem Cytochem.

[9] Goveia J, Stapor P, Carmeliet P (2014). Principles of targeting endothelial cell metabolism to treat angiogenesis and endothelial cell dysfunction in disease. EMBO Mol. Med..

[10] Schoors S (2014). Partial and transient reduction of glycolysis by PFKFB3 blockade reduces pathological angiogenesis. Cell Metab..

[11] De Bock K (2013). Role of PFKFB3-driven glycolysis in vessel sprouting. Cell.

[12] Siemerink MJ (2012). CD34 marks angiogenic tip cells in human vascular endothelial cell cultures. Angiogenesis.

[13] del Toro R (2010). Identification and functional analysis of endothelial tip cell-enriched genes. Blood.

[14] Strasser GA, Kaminker JS, Tessier-Lavigne M (2010). Microarray analysis of retinal endothelial tip cells identifies CXCR4 as a mediator of tip cell morphology and branching. Blood.

[15] Harrington LS (2008). Regulation of multiple angiogenic pathways by Dll4 and Notch in human umbilical vein endothelial cells. Microvasc. Res..

[16] Dallinga MG (2018). IGF2 and IGF1R identified as novel tip cell genes in primary microvascular endothelial cell monolayers. Angiogenesis.

[17] Stapor P, Wang X, Goveia J, Moens S, Carmeliet P (2014). Angiogenesis revisited - role and therapeutic potential of targeting endothelial metabolism. J. Cell Science.

[18] Psychogios N (2011). The human serum metabolome. PloS One.

[19] Larsson A, Sköldenberg E, Ericson H (2002). Serum and plasma levels of FGF-2 and VEGF in healthy blood donors. Angiogenesis.

[20] van Duinen V (2019). Perfused 3D angiogenic sprouting in a high-throughput in vitro platform. Angiogenesis.

[21] Divakaruni AS, Paradyse A, Ferrick DA, Murphy AN, Jastroch M (2014). Analysis and interpretation of microplate-based oxygen consumption and pH data. Methods Enzymol..

[22] Gerhardt H (2003). VEGF guides angiogenic sprouting utilizing endothelial tip cell filopodia. J. Cell Biol..

[23] Siemerink MJ, Augustin AJ, Schlingemann RO (2010). Mechanisms of ocular angiogenesis and its molecular mediators. Dev Ophthalmol.

[24] Sainson RC (2008). TNF primes endothelial cells for angiogenic sprouting by inducing a tip cell phenotype. Blood.

[25] Koon YL, Zhang S, Rahmat MB, Koh CG, Chiam K-H (2018). Enhanced Delta-Notch Lateral Inhibition Model Incorporating Intracellular Notch Heterogeneity and Tension-Dependent Rate of Delta-Notch Binding that Reproduces Sprouting Angiogenesis Patterns. Sci. Rep..

[26] Spuul P (2016). VEGF-A/Notch-Induced Podosomes Proteolyse Basement Membrane Collagen-IV during Retinal Sprouting Angiogenesis. Cell Rep..

[27] Tennant DA, Duran RV, Boulahbel H, Gottlieb E (2009). Metabolic transformation in cancer. Carcinogenesis.

[28] Kalucka J (2015). Metabolic control of the cell cycle. Cell cycle.

[29] Wu G, Haynes TE, Li H, Meininger CJ (2000). Glutamine metabolism in endothelial cells: ornithine synthesis from glutamine via pyrroline-5-carboxylate synthase. Comp. Biochem. Physiol. A Mol. Integr. Physiol..

[30] Harjes U, Bensaad K, Harris AL (2012). Endothelial cell metabolism and implications for cancer therapy. Br. J. Cancer.

[31] Polet F, Feron O (2013). Endothelial cell metabolism and tumour angiogenesis: glucose and glutamine as essential fuels and lactate as the driving force. J. Intern. Med..

[32] Hinshaw DB, Burger JM (1990). Protective effect of glutamine on endothelial cell ATP in oxidant injury. J. Surg. Res..

[33] Schoors S (2015). Fatty acid carbon is essential for dNTP synthesis in endothelial cells. Nature.

[34] Dagher Z, Ruderman N, Tornheim K, Ido Y (2001). Acute regulation of fatty acid oxidation and amp-activated protein kinase in human umbilical vein endothelial cells. Circ Res.

[35] Coutelle O (2014). Embelin inhibits endothelial mitochondrial respiration and impairs neoangiogenesis during tumor growth and wound healing. EMBO Mol Med.

[36] Vandekeere S (2018). Serine Synthesis via PHGDH Is Essential for Heme Production in Endothelial Cells. Cell Metab..

[37] Diebold L, Chandel NS (2016). Mitochondrial ROS regulation of proliferating cells. Free Radic. Biol. Med..

[38] Dewhirst MW (2009). Relationships between cycling hypoxia, HIF-1, angiogenesis and oxidative stress. Radiation Res..

[39] Daneau G, Boidot R, Martinive P, Feron O (2010). Identification of cyclooxygenase-2 as a major actor of the transcriptomic adaptation of endothelial and tumor cells to cyclic hypoxia: effect on angiogenesis and metastases. Clin. Cancer Res..

[40] Huang H (2017). Role of glutamine and interlinked asparagine metabolism in vessel formation. EMBO J..

[41] Gerhardt H (2008). VEGF and endothelial guidance in angiogenic sprouting. Organogenesis.

[42] Yeh WL, Lin CJ, Fu WM (2008). Enhancement of glucose transporter expression of brain endothelial cells by vascular endothelial growth factor derived from glioma exposed to hypoxia. Mol. Pharmacol..

[43] Parra-Bonilla G, Alvarez DF, Al-Mehdi AB, Alexeyev M, Stevens T (2010). Critical role for lactate dehydrogenase A in aerobic glycolysis that sustains pulmonary microvascular endothelial cell proliferation. Am. J. Physiol. Lung Cell Mol. Physiol..

[44] Sun K (2014). Brown adipose tissue derived VEGF-A modulates cold tolerance and energy expenditure. Mol. Metab..

[45] Austin S, St-Pierre J (2012). PGC1alpha and mitochondrial metabolism–emerging concepts and relevance in ageing and neurodegenerative disorders. J. Cell Sci..

[46] Jakobsson L (2010). Endothelial cells dynamically compete for the tip cell position during angiogenic sprouting. Nat. Cell Biol..

[47] Nowak-Sliwinska P (2018). Consensus guidelines for the use and interpretation of angiogenesis assays. Angiogenesis.

[48] Seguin F (2012). The fatty acid synthase inhibitor orlistat reduces experimental metastases and angiogenesis in B16-F10 melanomas. Br. J. Cancer.

[49] Cruys B (2016). Glycolytic regulation of cell rearrangement in angiogenesis. Nat. Commun..

[50] Eklund L, Bry M, Alitalo K (2013). Mouse models for studying angiogenesis and lymphangiogenesis in cancer. Mol. Oncol..

[51] Crampton, S. P., Davis, J. & Hughes, C. C. Isolation of human umbilical vein endothelial cells (HUVEC). *JoVE* **183**, 10.3791/183 (2007). 10.3791/183 PMC2576276 18978951

[52] Van Hinsbergh VW, Sprengers ED, Kooistra T (1987). Effect of thrombin on the production of plasminogen activators and PA inhibitor-1 by human foreskin microvascular endothelial cells. Thromb. Haemost..

[53] Preibisch S, Saalfeld S, Tomancak P (2009). Globally optimal stitching of tiled 3D microscopic image acquisitions. Bioinformatics.

[54] Zengel P (2011). μ-Slide Chemotaxis: A new chamber for long-term chemotaxis studies. BMC Cell Biology.

[55] Ferrick DA, Neilson A, Beeson C (2008). Advances in measuring cellular bioenergetics using extracellular flux. Drug Discov. Today.

[56] Klaassen I (2009). Altered expression of genes related to blood-retina barrier disruption in streptozotocin-induced diabetes. Exp. Eye Res..

[57] Andersen CL, Jensen JL, Orntoft TF (2004). Normalization of real-time quantitative reverse transcription-PCR data: a model-based variance estimation approach to identify genes suited for normalization, applied to bladder and colon cancer data sets. Cancer Res..

[58] Ruijter JM (2006). Factor correction as a tool to eliminate between-session variation in replicate experiments: application to molecular biology and retrovirology. Retrovirology.

