# Supplementary material for: Endothelial tip cells in vitro are less glycolytic and have a more flexible response to metabolic stress than non-tip cells
Source: Sci Rep. 2019 Jul 18;9:10414. doi: 10.1038/s41598-019-46503-2 (PMC6639367; doi:10.1038/s41598-019-46503-2)
Supplement: Supplementary file 1 — Supplementary info [file 41598_2019_46503_MOESM1_ESM.pdf]

Endothelial tip cells *in vitro* are less glycolytic and have a more flexible response to metabolic stress than non-tip cells

B. Yetkin-Arik [1,2], I.M.C. Vogels [1,2], N. Neyazi [1,2], V. van Duinen [3,4], R.H. Houtkooper [5], C.J.F. van Noorden [2,6], I. Klaassen [1,2#*], R.O. Schlingemann [1,7#]

[1] Ocular Angiogenesis Group, Department of Ophthalmology and [2] Department of Medical Biology, Amsterdam Cardiovascular Sciences, Cancer Center Amsterdam, Amsterdam UMC, University of Amsterdam, Meibergdreef 9, Amsterdam, The Netherlands.

[3] Department of Systems Biomedicine and Pharmacology, Leiden University, Leiden, The Netherlands.

[4] Department of Internal Medicine, Division of Nephrology and the Einthoven Laboratory for Vascular and Regenerative Medicine, Leiden University Medical Centre, Leiden, The Netherlands.

[5] Laboratory Genetic Metabolic Diseases, Amsterdam Gastroenterology and Metabolism, Amsterdam Cardiovascular Sciences, Amsterdam UMC, University of Amsterdam, Meibergdreef 9, Amsterdam, The Netherlands.

[6] Department of Genetic Toxicology and Cancer Biology, National Institute of Biology, Ljubljana, Slovenia.

[7] Department of Ophthalmology, University of Lausanne, Jules-Gonin Eye Hospital, Fondation Asile des Aveugles, Lausanne, Switzerland.

[#] Authors contributed equally.

* Correspondence:

Dr. I. Klaassen, Department of Medical Biology, Amsterdam UMC, Academic Medical Center, Meibergdreef 15, Room L3-154, 1105 AZ Amsterdam, The Netherlands. E-mail: i.klaassen@amc.uva.nl.

Supplementary information

Supplementary Table 1. Primer details. Gene symbols, primer sequences, predicted size (bp) and melting temperature (Tm) of the amplification products for human genes tested.

Gene	GenBank	Forward primer	Reverse primer	Size(bp)	Tm (°C)
**ANGPT2**	NM_001147	GCAAAATAAGCAGCATCAGCCAAC	GCATCAAACCACCAGCCTCCT	115	76
**CD34**	NM_001025109	GGAGCAGGCTGATGCTGATG	ATCCCCAGCTTTTTCAGGTCAGAT	165	82
**CXCR4**	NM_003467.2	CGGTTACCATGGAGGGGATCAGTATATA	GCATTTTCTTCACGGAAACAGGGTTC	109	78
**DLL4**	NM_019074	AACGGGGGACAGTGCCTGAA	TGAGCCCATTCTCCAGGTCAT	151	86
**IGF2**	NM_000612.4	CCTCGTGCTGCATTGCTGCT	CTTGCGGGCCTGCTGAAGTAGAA	115	86
**NRP2**	NM_003872	GGAGCCCTGTGGTTGGATGTATG	TCATCTGGAAACGTCCGGTCGT	93	83
**VEGFR2**	NM_002253.2	CCAGATGACAACCAGACGGACAG	GGCACCATTCCACCAAAAGATG	104	77
**VEGFR3**	NM_002020	GCCAGGTATTACAACTGGGTGTCCT	TCTGGTTGTCCACAGAGCCTTTGT	136	83
**YWHAZ**	NM_003406	ACTTTTGGTACATTGTGGCTTCAA	CCGCCAGGACAAACCAGTAT	94	77

Supplementary Table 2. Primer details. Gene symbols, primer sequences, and location of human genes tested to determine mitochondrial DNA and nuclear DNA content.

Gene	GenBank	Forward primer	Reverse primer	Cellular location
**COX2**	NC_011137.1	GATCCCTCCCTTACCATCAAA	GCCGTAGTCGGTGTACTCGT	Mitochondrial DNA
**COX3**	NC_012920.1	TCCAAACATCACTTTGGCTTC	AACCACATCTACAAAATGCCAGT	Mitochondrial DNA
**CYTB**	NC_011137.1	TCATTATTGCAGCCCTAGCAG	GTTGTTTGATCCCGTTTCGT	Mitochondrial DNA
**NCOA3**	NC_000020.11	CCTCTGGGCTTTTATTGCGAC	CGGTCATCAGAAGAACAGGTAAGT	Nuclear DNA
**B2M**	NC_000015.10	AAGTTCGCATGTCCTAGCACC	TCACAGCCAAGCATTCTACAAAC	Nuclear DNA

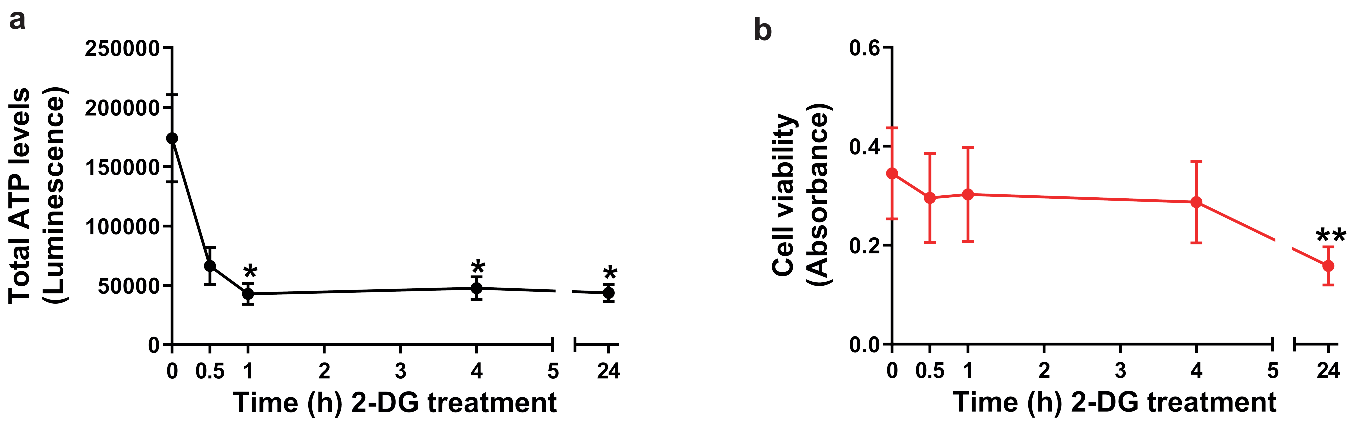

Fig. S1 Effects of blocking cellular glucose uptake on energy production and survival of HUVECs and on tip cell differentiation.

a HUVECs in culture were exposed to 100 mM 2-DG for 0.5, 1, 4, or 24 h and showed rapid depletion of total ATP levels during the first hour of treatment in HUVECs (determined as relative luminescence). Total ATP levels remained low up to 24 h of exposure. **b** HUVECs treated with 2-DG showed a significantly reduced viability (measured as absorbance) in the MTT assay after 24 h of treatment. Results are shown as means ± SEM of experiments with HUVECs of at least 3 donors. * P<0.05 and ** P<0.01 as compared to control (Unpaired Student's t-test).

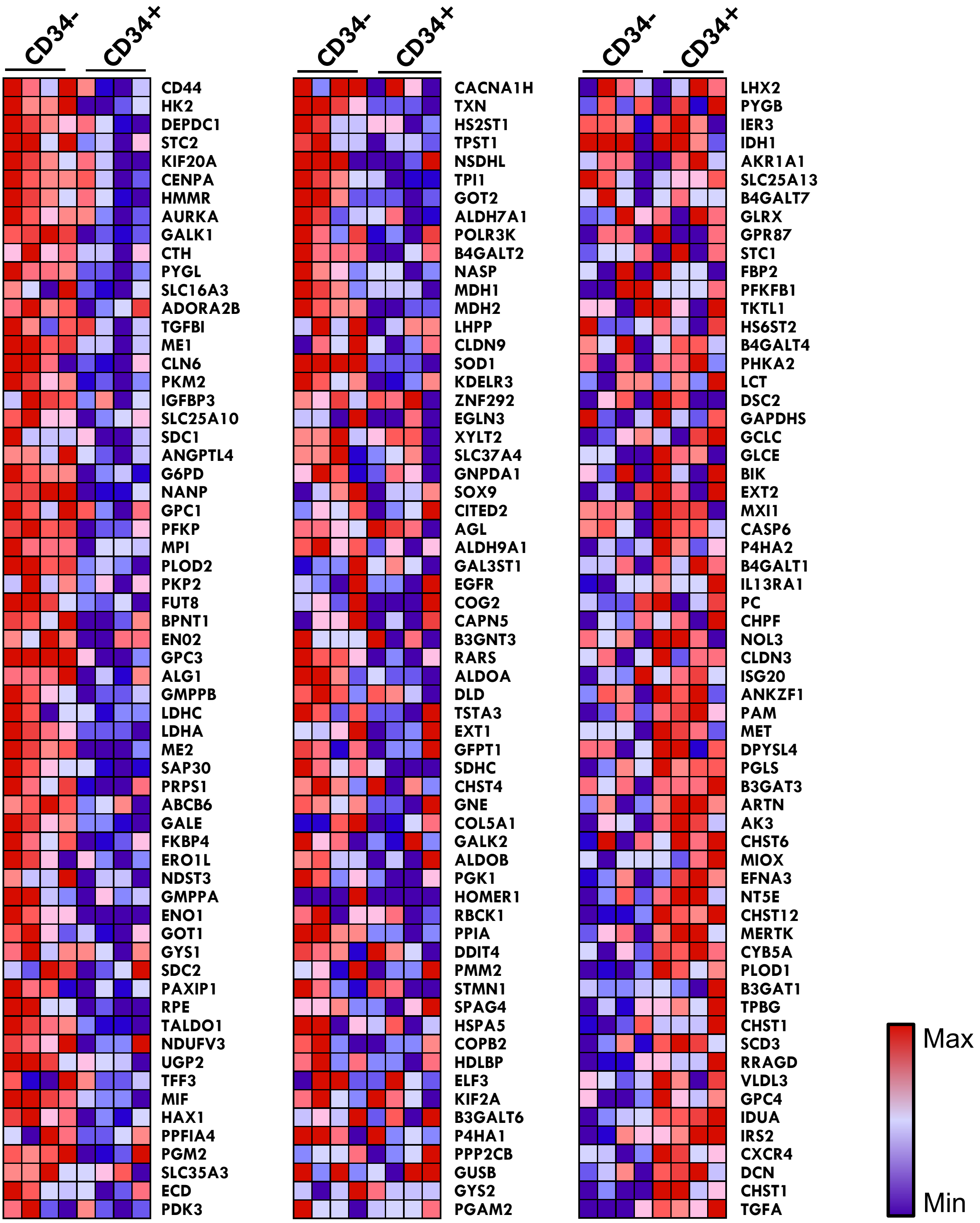

Fig. S2 Heat map differentially expressed genes involved in the glycolytic pathway in non-tip cells and tip cells. Heat map of the accompanying GSEA enrichment plot (Fig. 4d) for the glycolysis gene set in CD34⁻ non-tip cells and CD34⁺ tip cells are shown (N=4).

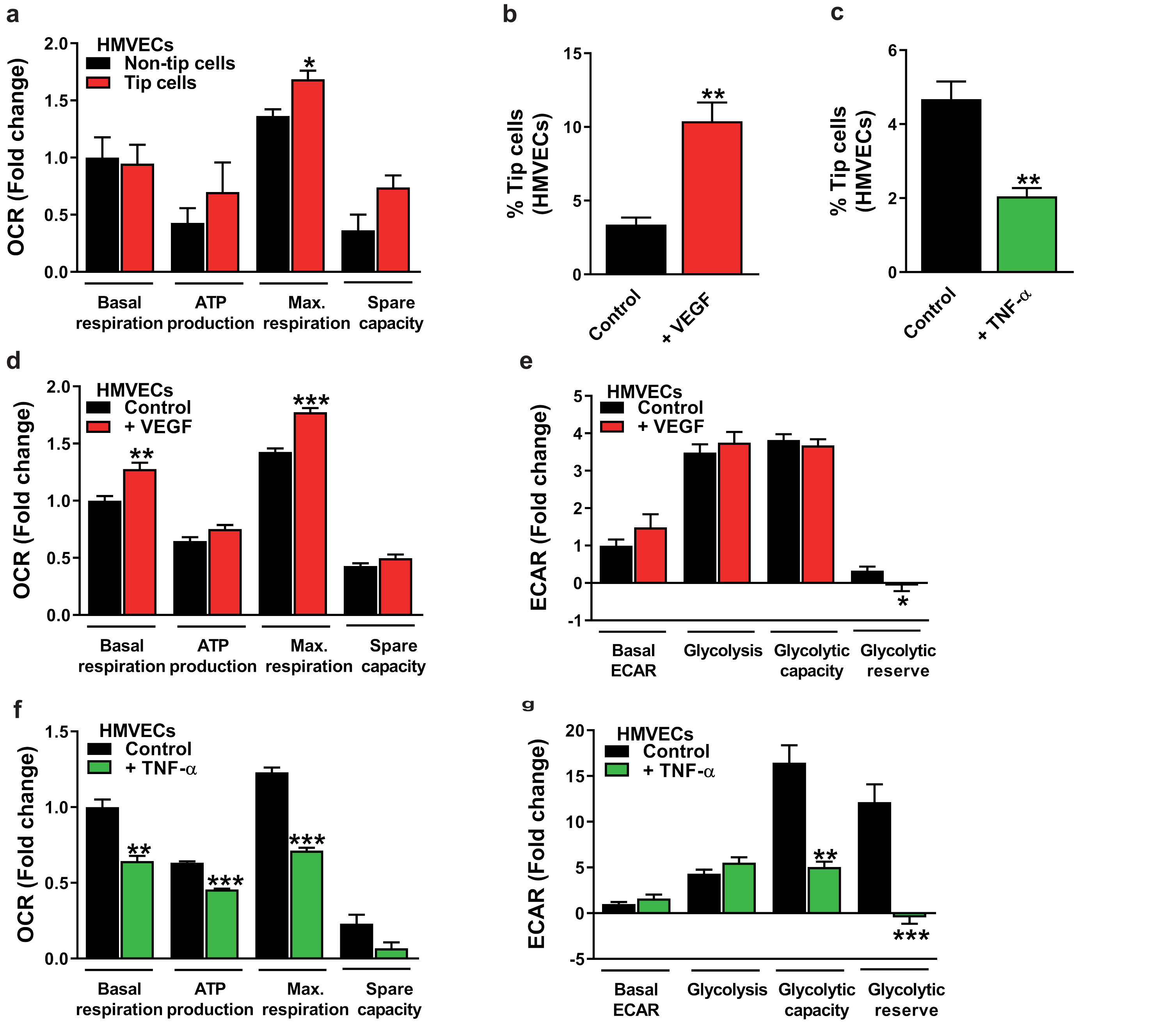

Fig. S3 Mitochondrial respiration and glycolysis in hMVECs.
HMVEC cells were treated with VEGF (25 ng/ml) or TNF-α (10 ng/ml) for 24 h. **a** FACS-sorted hMVECs showed a higher maximal respiration capacity and spare capacity in tip cells as compared to non-tip cells. Treatment of hMVEC cultures with VEGF induced the percentage of tip cells (**b**), whereas TNF-α reduced the percentage of tip cells (**c**). VEGF induced mitochondrial respiration (**d**) and reduced glycolytic reserve (**e**). TNF-α reduced mitochondrial respiration (**f**) and glycolytic capacity and glycolytic reserve (**g**). OCR and ECAR measurements were represented as fold change compared to control basal OCR and ECAR levels, respectively. Results are shown as means ± SEM of experiments with hMVECs of at least 3 donors. * P<0.05, **bP<0.01, and *** P<0.001 as compared to control (Unpaired Student's t-test).

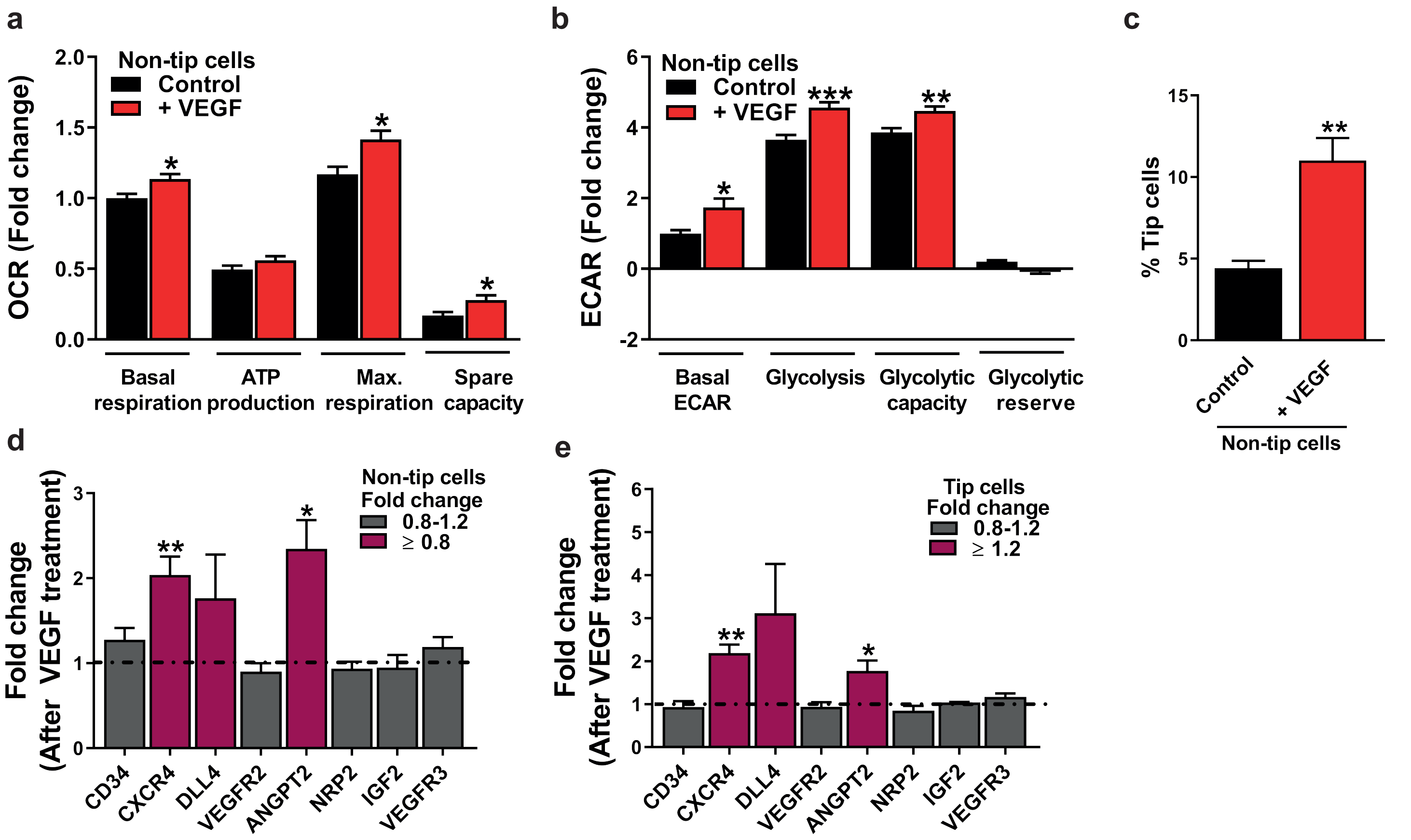

Fig. S4 Effects of VEGF on mitochondrial respiration, glycolysis and tip cell differentiation.
HUVECs were treated with VEGF (25 ng/ml) for 24 h. VEGF induced mitochondrial respiration (**a**) and glycolysis (**b**) in non-tip cells. Non-tip cells treated with VEGF showed induced percentages of tip cells (**c**) and induced mRNA expression levels of CXCR4, DLL4, and ANGPT2 (**d**). **e** Tip cells treated with VEGF did not show reduced mRNA expression levels of tip cell-specific genes. OCR and ECAR measurements were represented as fold change compared to control basal OCR and ECAR levels, respectively. Results are shown as means ± SEM of experiments with HUVECs of at least 3 donors. * $P<0.05$, ** $P<0.01$, and *** $P<0.001$ as compared to control (Unpaired Student's t-test).

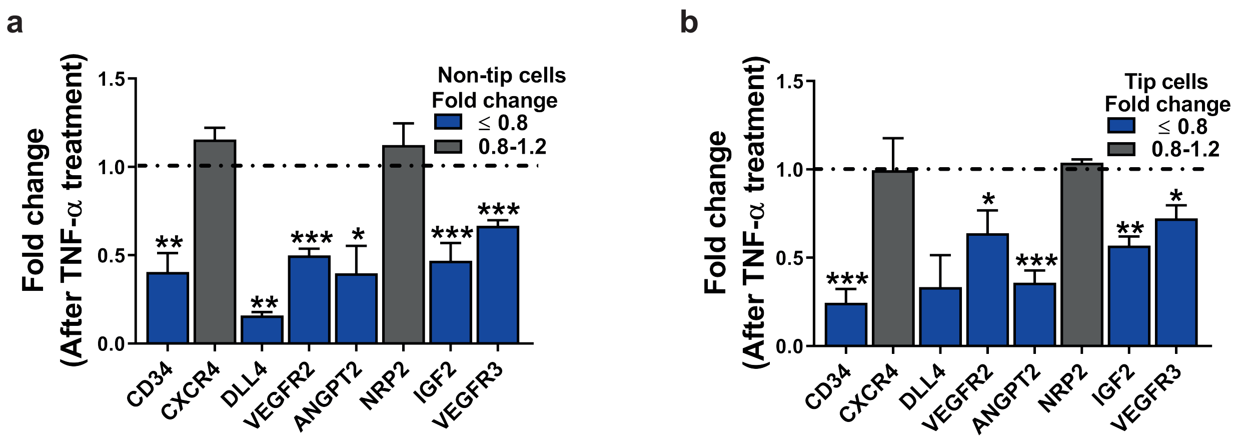

Fig. S5 Effects of TNF-α on mRNA expression levels of tip cell-specific genes.
Treatment of FACS-sorted HUVECs with TNF-α (10 ng/ml) reduced mRNA expression levels of 6 out of 8 tip cell-specific genes in non-tip cells (**a**) and reduced mRNA expression levels of 5 out of 8 tip cell-specific genes in tip cells (**b**) at 24 h after treatment. Results are shown as means ± SEM of experiments with HUVECs of at least 3 donors. * $P<0.05$, ** $P<0.01$, and *** $P<0.001$ as compared to control (Unpaired Student's t-test).

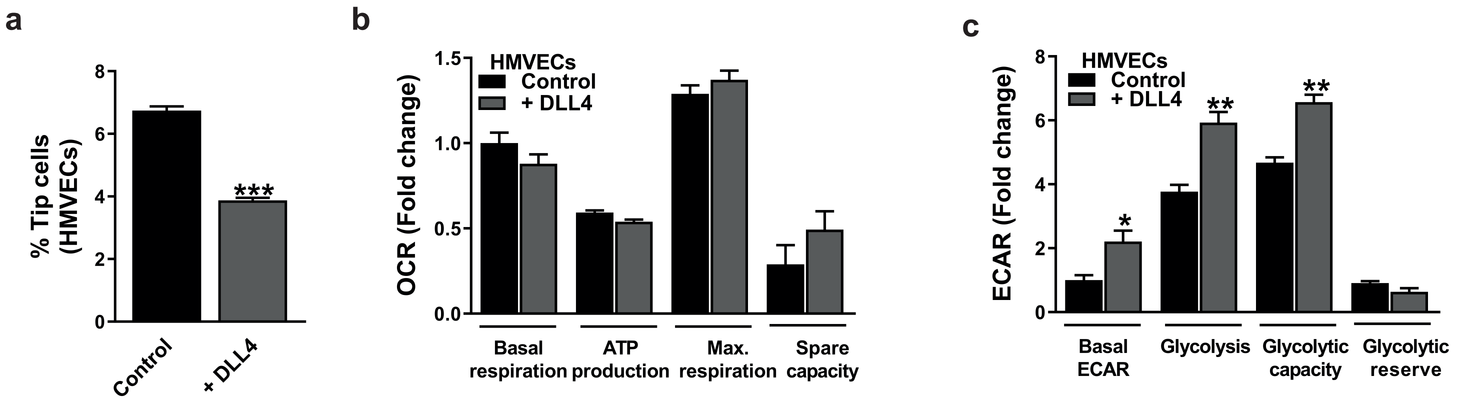

Fig. S6 Effects of DLL4 coating on mitochondrial respiration and glycolysis in hMVECs.
a HMVECs cultured on DLL4-coated (1µg/ml) plates showed lower tip cell percentages compared to BSA-coated (1 µg/ml) plates used as a control. DLL4 treatment did not affect mitochondrial respiration (**b**), but increased glycolysis (**c**) in hMVEC cultures 24 h after cell addition. OCR and ECAR measurements were represented as fold change compared to control basal OCR and ECAR levels, respectively. Results are shown as means ± SEM of experiments with HUVECs of at least 3 donors. * $P<0.05$ and ** $P<0.01$ as compared to control (Unpaired Student's t-test).
